# ESIPT-Capable 4-(2-Hydroxyphenyl)-2-(Pyridin-2-yl)-1*H*-Imidazoles with Single and Double Proton Transfer: Synthesis, Selective Reduction of the Imidazolic OH Group and Luminescence

**DOI:** 10.3390/molecules28041793

**Published:** 2023-02-14

**Authors:** Nikita A. Shekhovtsov, Elena B. Nikolaenkova, Alexey A. Ryadun, Denis G. Samsonenko, Alexsei Ya. Tikhonov, Mark B. Bushuev

**Affiliations:** 1 Nikolaev Institute of Inorganic Chemistry, Siberian Branch of Russian Academy of Sciences, 3, Acad. Lavrentiev Ave., Novosibirsk 630090, Russia; 2 N. N. Vorozhtsov Novosibirsk Institute of Organic Chemistry, Siberian Branch of Russian Academy of Sciences, 9, Acad. Lavrentiev Ave., Novosibirsk 630090, Russia

**Keywords:** imidazole, 2-hydroxyphenyl group, hydrogen bond, ESIPT, luminescence

## Abstract

1*H*-Imidazole derivatives establish one of the iconic classes of ESIPT-capable compounds (ESIPT = excited state intramolecular proton transfer). This work presents the synthesis of 1-hydroxy-4-(2-hydroxyphenyl)-5-methyl-2-(pyridin-2-yl)-1*H*-imidazole (**L^OH,OH^**) as the first example of ESIPT-capable imidazole derivatives wherein the imidazole moiety simultaneously acts as a proton acceptor and a proton donor. The reaction of **L^OH,OH^** with chloroacetone leads to the selective reduction of the imidazolic OH group (whereas the phenolic OH group remains unaffected) and to the isolation of 4-(2-hydroxyphenyl)-5-methyl-2-(pyridin-2-yl)-1*H*-imidazole (**L^H,OH^**), a monohydroxy congener of **L^OH,OH^**. Both **L^OH,OH^** and **L^H,OH^** demonstrate luminescence in the solid state. The number of OH···N proton transfer sites in these compounds (one for **L^H,OH^** and two for **L^OH,OH^**) strongly affects the luminescence mechanism and color of the emission: **L^H,OH^** emits in the light green region, whereas **L^OH,OH^** luminesces in the orange region. According to joint experimental and theoretical studies, the main emission pathway of both compounds is associated with T_1_ → S_0_ phosphorescence and not related to ESIPT. At the same time, **L^OH,OH^** also exhibits S_1_ → S_0_ fluorescence associated with ESIPT with one proton transferred from the hydroxyimidazole moiety to the pyridine moiety, which is not possible for **L^H,OH^** due to the absence of the hydroxy group in the imidazole moiety.

## 1. Introduction

**Scheme 1:**
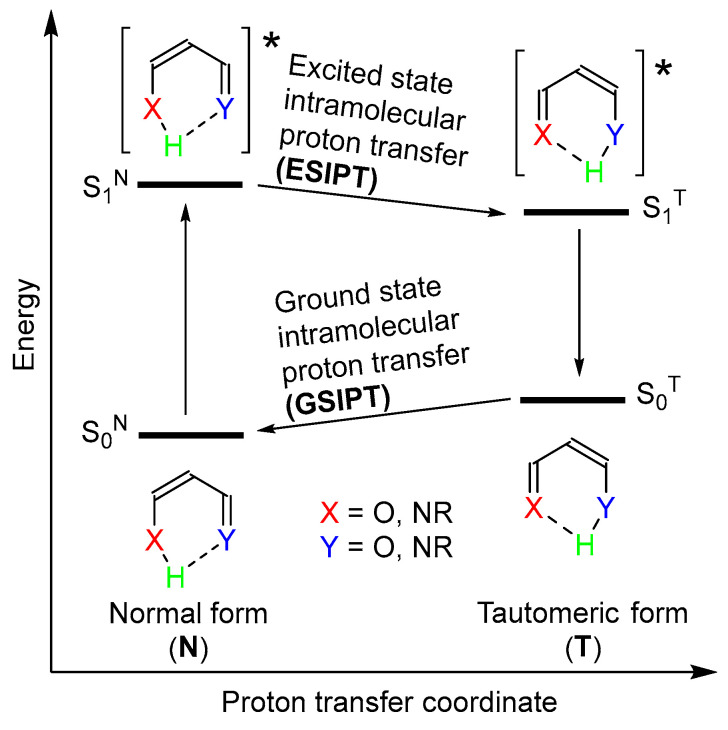
The ESIPT and GSIPT processes in molecules featuring short intramolecular hydrogen bonds.

Aromatic and heteroaromatic compounds featuring strong intramolecular hydrogen bonds of the O–H···Y and N–H···Y types (Y = O, NR) can manifest photoinduced intramolecular proton transfer reactions (Scheme 1) [1,2,3,4,5,6,7,8,9,10,11,12,13,14]. The photoexcitation of such molecules in their most stable, or normal (N), form leads to the electron density redistribution, followed by the excited state intramolecular proton transfer (ESIPT) reaction yielding the excited state tautomeric form (T). Radiative and non-radiative processes proceeding in the tautomeric form convert this excited state form into the ground state. The last step in this sequence of processes is the ground state intramolecular proton transfer (GSIPT) reaction, converting the tautomeric form to the normal one.

The ESIPT photoreaction (Scheme 1) is highly sensitive to substituents [15,16,17,18,19,20,21,22,23,24,25,26,27] and coordinated metal ions [28,29,30,31,32,33], protonation/deprotonation [34,35,36,37,38,39,40,41], the state of aggregation [42,43], the polarity of solvent [44,45,46,47,48] and the presence of various analytes [49,50,51,52,53,54,55,56]. If the excited state tautomerization (normal-to-tautomeric) is barrierless, the only form to emit is the tautomeric one, which typically luminesces with rather large Stokes shift [57,58]. In the case of barriers on excited state potential energy surfaces, the molecule can be trapped in a local minimum of the normal form, leading to the emission of the normal form. Modifying the barrier height in the excited state, one can achieve dual emission associated with the luminescence of both forms [59,60,61,62,63,64,65,66]. The sensitivity of ESIPT-capable compounds to various stimuli makes them an appealing platform for numerous applications [67,68,69].

1*H*-Imidazoles, 1,3-oxazoles, 1,3-thiazoles and their benzannulated congeners are often used in the design of ESIPT-fluorophores [70,71,72,73,74,75,76,77,78,79,80,81,82]. Normally, when decorated with such proton-donating groups as unsubstituted or substituted 2-hydroxyphenyl groups in the α-position to aza-atoms, their free nitrogen atoms act as proton acceptors during the ESIPT process [70,71,72,73,74,75,76,77,78,79,80,81,82]. Recently we proposed a new approach in the design of imidazole-based ESIPT-fluorophores in which we switched the role of the imidazole cycle to the one of a proton donor by introducing the hydroxy group in the position 1 and the pyridin-2-yl group in the position 2 of the imidazole ring [83,84,85,86,87]. Importantly, both roles of the imidazole ring in ESIPT-fluorophores, i.e., the proton acceptor and the proton donor ones, can be combined in a single molecule if we introduce the proton-donating 2-hydroxyphenyl group in the position 4 and the proton accepting pyridin-2-yl group in the position 2 of the 1-hydroxy-1*H*-imidazole moiety. In this case, the molecule will feature two spatially separated ESIPT-sites with two short O–H···N hydrogen bonds therein.

**Scheme 2:**
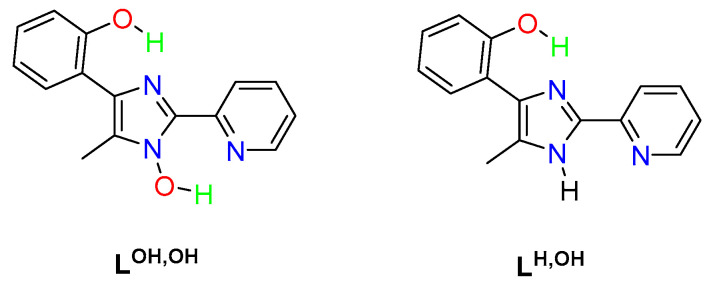
Structural formulae of **L^OH,OH^** and **L^H,OH^**.

In this manuscript, we report the synthesis of 1-hydroxy-4-(2-hydroxyphenyl)-5-methyl-2-(pyridin-2-yl)-1*H*-imidazole (**L^OH,OH^**) as the first example of imidazole derivatives wherein the central 1-hydroxy-1*H*-imidazole moiety simultaneously acts both as a proton acceptor and a proton donor (Scheme 2). Along with the synthesis of **L^OH,OH^**, we report the reaction of **L^OH,OH^** with chloroacetone leading to the selective formation of a corresponding 1*H*-imidazole derivative, 4-(2-hydroxyphenyl)-5-methyl-2-(pyridin-2-yl)-1*H*-imidazole (**L^H,OH^**) (Scheme 2), and proceeding without affecting the phenolic hydroxy group. Finally, we present the results of combined comparative experimental and theoretical studies of the emission of **L^OH,OH^** and **L^H,OH^** and the ESIPT photoreactions in both compounds.

## 2. Results and Discussion

### 2.1. Synthesis of 1-Hydroxy-4-(2-Hydroxyphenyl)-5-Methyl-2-(Pyridin-2-yl)-1H-Imidazole (**L^OH,OH^**) and 4-(2-Hydroxyphenyl)-5-Methyl-2-(Pyridin-2-yl)-1H-Imidazole (**L^H,OH^**)

**Scheme 3:**
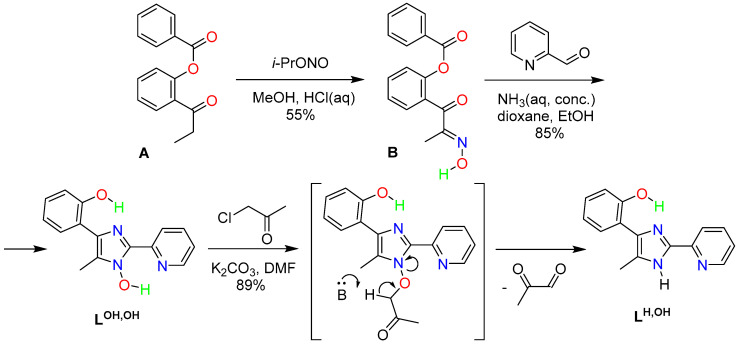
Synthesis of **L^OH,OH^** and **L^H,OH^**.

The ESIPT-capable imidazole-based compounds **L^OH,OH^** and **L^H,OH^** were synthesized using the following reactions (Scheme 3). The first step, i.e., the nitrosation reaction, required the protection of the hydroxy group in *ortho* hyroxypropiophenone with the benzoyl group [88]. After this, the monoxime **B** was prepared by the nitrosation of 2-benzoyloxypropiophenone (**A**) with isopropyl nitrite according to the procedure close to the one reported by Mason [88]. The second step was the construction of the 1-hydroxy-1*H*-imidazole moiety. The most convenient and widespread method for the synthesis of 1-hydroxy-1*H*-imidazoles is the condensation of monoxime diketones with aldehydes and ammonia or ammonium acetate [89]. The condensation of the monoxime **B** with pyridinecarboxaldehyde and ammonia (cf. [83]) led to the isolation of 1-hydroxy-4-(2-hydroxyphenyl)-5-methyl-2-(pyridin-2-yl)-1*H*-imidazole (**L^OH,OH^**). Importantly, the benzoyl protecting group removal occurred at this step along with the simultaneous formation of the imidazole ring. The last step was the conversion of the 1-hydroxy-1*H*-imidazole derivative **L^OH,OH^** to the 1*H*-imidazole **L^H,OH^**. For this conversion, along with various reducing agents (e.g., PCl_3_, (Ph)_3_P, trialkylphosphites, TiCl_3_, etc.), halogen-substituted compounds with electron-withdrawing groups (e.g., BrCH_2_CO_2_Me [90] and chloroacetone [91,92]) can be used. The interaction of 1-hydroxy-1*H*-imidazole with chloroacetone allows the reaction to be carried out under mild conditions through the intermediate formation of a chlorine atom substitution product, followed by its fragmentation to form reduced 1*H*-imidazole. Importantly, the reaction of **L^OH,OH^** with chloroacetone (cf. [93]) proceeded without affecting the phenolic hydroxy group, which greatly simplified the preparation of the 1*H*-imidazole **L^H,OH^** compound. Spectral and structural data for the compounds are given in Supplementary Materials.

### 2.2. X-ray Single Crystal Structure of 1-Hydroxy-4-(2-Hydroxyphenyl)-5-Methyl-2-(Pyridin-2-yl)-1H-Imidazole (**L^OH,OH^**)

**Figure 1:**
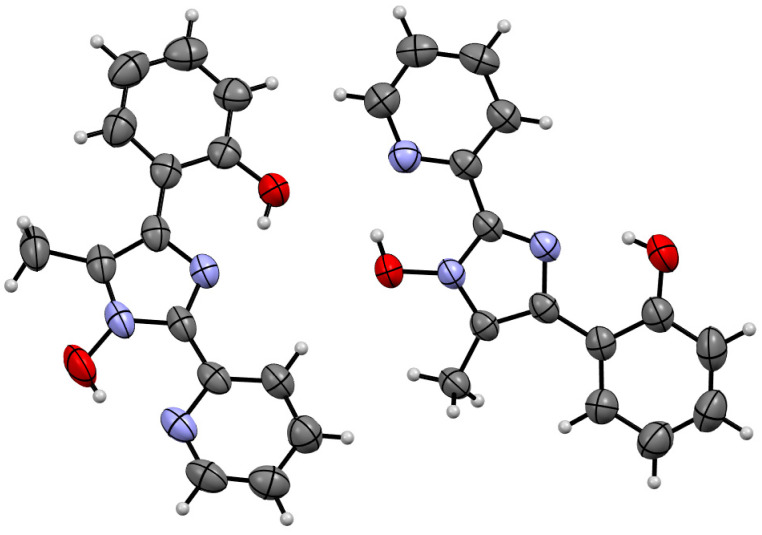
Two crystallographically independent molecules in the structure of **L^OH,OH^**.

The dihydroxy derivative, **L^OH,OH^**, crystallizes in the monoclinic space group *P*2_1_/c (Supplementary Materials, Table S1, Figures S9–S11). There are two crystallographically independent **L^OH,OH^** molecules in the crystal structure (Figure 1). The 2-(pyridin-2-yl)imidazole moiety in both independent molecules is practically planar with the torsions smaller than 1°. On the other hand, the 4-(2-hydroxyphenyl) group deviates from the plane of the imidazole cycle by *ca*. 7° in one and by *ca*. 12° in another **L^OH,OH^** molecule. There are two short intramolecular O–H···N hydrogen bonds in each molecule with the O···N separations of 2.57–2.60 Å.

**Figure 2:**
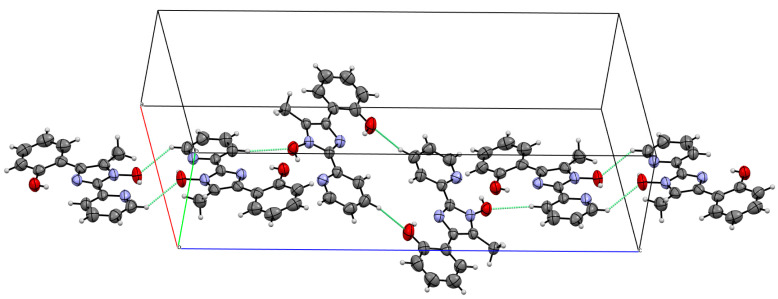
A supramolecular ribbon in the structure of **L^OH,OH^**.

The **L^OH,OH^** molecules are assembled into corrugated ribbons running along the *c* axis through weak C–H···O hydrogen bonds (Figure 2). The ribbons are further gathered into 3D supramolecular structure via C–H···C and C–H···H–C van der Waals interactions (Supplementary Materials, Figures S9–S11). 

### 2.3. Tautomeric Forms of **L^H,OH^** and **L^OH,OH^**: An Introduction 

**Scheme 4:**
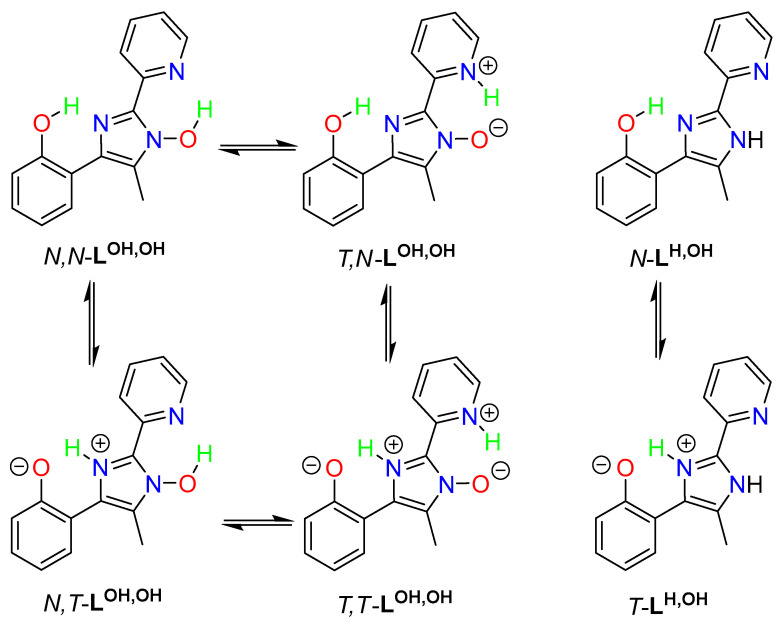
Tautomeric forms of **L^H,OH^** and **L^OH,OH^**.

**L^H,OH^** and **L^OH,OH^** can exist in various tautomeric forms. In this context, for the sake of clarity we introduce the following abbreviations of these forms for further discussions (Scheme 4). **L^OH,OH^** has two proton transfer sites and therefore can exist in four tautomeric forms: i) *N*,*N*-**L^OH,OH^** (no proton transferred, corresponds to the global energy minimum and to the X-ray crystal structure), (ii) *N*,*T*-**L^OH,OH^** (one proton transferred from the hydroxyphenyl moiety to the hydroxyimidazole moiety), (iii) *T*,*N*-**L^OH,OH^** (one proton transferred from the hydroxyimidazole moiety to the pyridine moiety), (iv) *T*,*T*-**L^OH,OH^** (both protons transferred). **L^H,OH^** has only one proton transfer site and can exist in two tautomeric forms: (i) *N*-**L^H,OH^** (no proton transferred) and (ii) *T*-**L^H,OH^** (one proton transferred). The same abbreviations are used for the energy minima of ground and excited states, e.g., S_0_^N,N^, S_1_^T,N^, T_1_^T^, etc.

### 2.4. Absorption Properties of **L^H,OH^** and **L^OH,OH^** in MeCN

**Figure 3:**
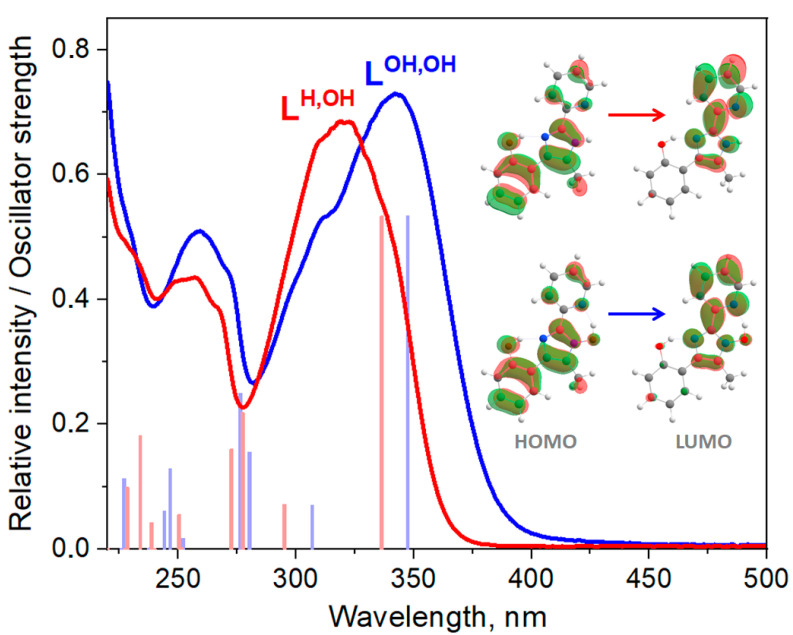
Absorption spectra of **L^H,OH^** (red) and **L^OH,OH^** (blue) in MeCN. Vertical bars display the positions and oscillator strengths of the singlet-to-singlet electronic transitions for **L^H,OH^** (red) and **L^OH,OH^** (blue).

In acetonitrile, both **L^H,OH^** and **L^OH,OH^** absorb in the ultraviolet domain, with the most intense peak centered at 320 and 342 nm, respectively (Figure 3). In order to test the relevance of the chosen theory level for quantum chemical computations, theoretical absorption spectra were calculated at the global energy minima of the ground state, S_0_^N,N^ (O^Ph^–H 0.988 Å, O^Imid^–H 1.010 Å, Table 1) for **L^OH,OH^** and S_0_^N^ (O^Ph^–H 0.980 Å) for **L^OH,OH^**. The energies and relative intensities of the calculated vertical singlet-to-singlet absorptions are in good agreement with the experimental data (Figure 3), showing the relevance of the functional and basis set used in this study. The most intense experimental peak corresponds to the first vertical singlet-to-singlet transition (S_0_ → S_1_), computed at 336 nm for **L^H,OH^** and 348 nm for **L^OH,OH^**. In accordance with the experimental spectra, this transition indeed has the highest oscillator strength (*ca.* 0.5) among the other transitions. In terms of molecular orbitals, S_0_ → S_1_ is a HOMO → LUMO transition. For both **L^H,OH^** and **L^OH,OH^**, HOMO is distributed over hydroxyphenyl and imidazole moieties, while LUMO is located on imidazole and pyridine moieties (Figure 3). Thus, the S_0_ → S_1_ absorption implies charge transfer from the hydroxyphenyl part of the molecule to the pyridine part. Despite there being no visual differences between the HOMO and LUMO of **L^H,OH^** and the HOMO and LUMO of **L^OH,OH^**, respectively, the most intensive absorption peak of **L^OH,OH^** is slightly red-shifted compared with that of **L^H,OH^**, and the computations fully reproduce this trend. A series of higher lying singlet-to-singlet transitions form the high-energy absorption band centered at *ca.* 260 nm for both ESIPT-emitters (Figure 3). 

**Figure 4:**
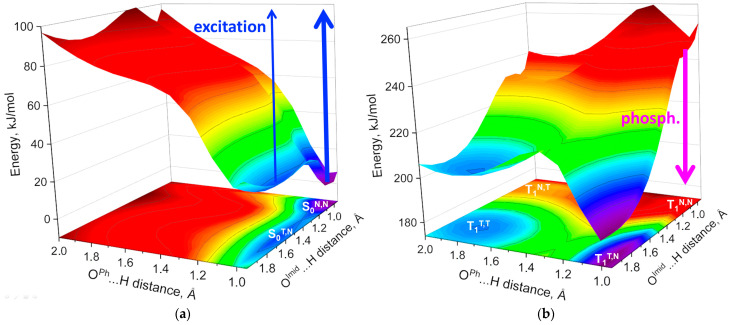
The potential energy surfaces (PESes) of the S_0_ (**a**) and T_1_ (**b**) states of **L^OH,OH^** along the proton transfer paths O^Ph^–H···N^Imid^ and O^Imid^–H···N^Py^ and their projections.

**Figure 5:**
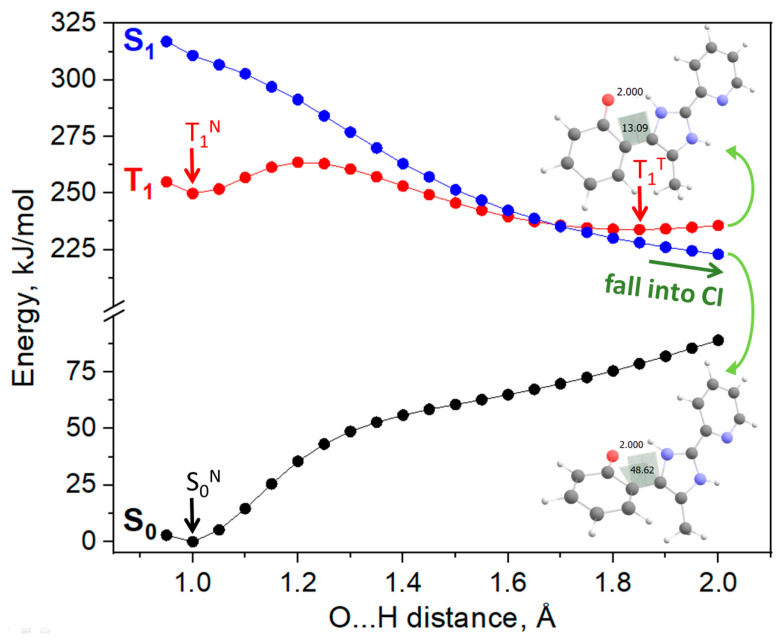
The potential energy curves (PECs) of the S_0_, S_1_ and T_1_ (right) states of **L^H,OH^** along the proton transfer path O^Ph^–H···N^Imid^. The arrows show the energy minima on these PECs. The optimized geometries of the T_1_ and S_1_ states with the O^Ph^–H distance of 2.0 Å are also depicted.

It is noteworthy that, in addition to the global energy minimum S_0_^N,N^ on the PES of the ground state, **L^OH,OH^** has a local minimum S_0_^T,N^ (O^Ph^–H 0.992 Å, O^Imid^–H 1.595 Å, Figure 4, Table 1), and therefore its corresponding form *T*,*N*-**L^OH,OH^** can also absorb light. S_0_^T,N^ is thermodynamically less favorable than S_0_^N,N^ by *ca.* 17 kJ/mol and is separated from S_0_^N,N^ by an energy barrier of *ca.* 20 kJ/mol. Although such a low barrier may indicate coexistence of the *N*,*N*-**L^OH,OH^** and *T*,*N*-**L^OH,OH^** tautomeric forms in solution, the fact that the experimental absorption spectrum is completely reproduced by the transitions of the *N*,*N*-**L^OH,OH^** form points to the very small contribution of the *T*,*N*-**L^OH,OH^** form to the absorption spectrum. In the case of **L^H,OH^**, there is only one minimum on the PEC of the ground state, S_0_^N^ (Figure 5).

### 2.5. Excitation and Emission Properties of **L^H,OH^** and **L^OH,OH^**

**Figure 6:**
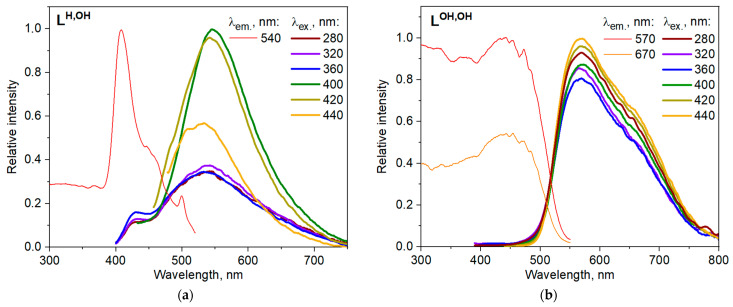
Excitation and emission spectra of **L^H,OH^** (**a**) and **L^OH,OH^** (**b**) in the solid state at room temperature.

**Figure 7:**
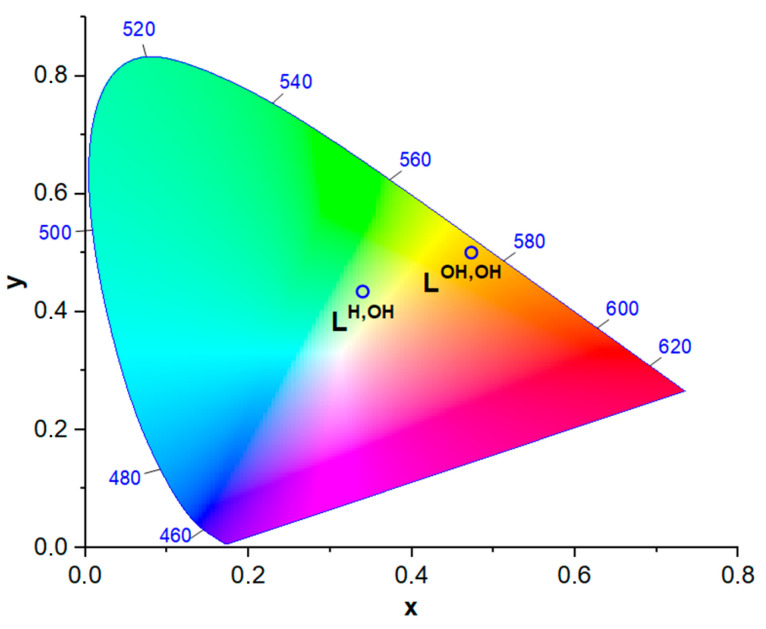
CIE 1931 diagram showing the chromaticity of the emission of **L^H,OH^** and **L^OH,OH^** in the solid state at λ_ex_ = 320 nm.

**L^H,OH^** and **L^OH,OH^** are non-luminescent in MeCN solution, indicating the possible predominance of various non-radiative deactivation pathways. In the solid state, **L^H,OH^** emits in the light green region (Figure 6 and Figure 7). The broad unstructured luminescence band of **L^H,OH^** is located in the region 400–750 nm with a maximum at 546 nm. The intensity of this band depends on excitation wavelength: at λ_ex_ = 400–420 nm, it is three times more intense than at λ_ex_ = 280–360 nm. However, a change in the excitation energy does not lead to a shift of the emission maximum. **L^H,OH^** exhibits a monoexponential photoluminescence decay (Supplementary Materials, Figure S14), indicating that there is likely only one emission mechanism. The lifetime of molecules in the excited state (τ) is 1.10 μs (λ_ex_ = 300 nm, λ_det_ = 540 nm), so the observed emission is associated with phosphorescence, i.e., with a spin-forbidden triplet-to-singlet transition. The width of the phosphorescence band is associated with the vibrational satellite structure, which involves an interplay of several transitions from the lowest vibrational level of the excited state to various vibrational levels of the ground state. 

**L^OH,OH^** demonstrates luminescence in the orange region (Figure 6 and Figure 7). As for **L^H,OH^**, the emission spectrum is dominated by a broad band at 450–800 nm centered at 568 nm. In contrast to **L^H,OH^**, an additional low-energy shoulder at *ca.* 670 nm appears in the case of **L^OH,OH^**, which is responsible for the orange color of luminescence. The emission band is more or less equally intensive when excited at λ_ex_ = 280–440 nm. The luminescence decay of **L^OH,OH^** is multiexponentional and more complex than for **L^H,OH^**: the long part of the photoluminescence decay reveals one lifetime in the microsecond range, τ = 1.05 μs (similar to **L^H,OH^**), whereas the short part reveals two lifetimes in the nanosecond range, τ = 2 ns and τ = 21 ns (Supplementary Materials, Figure S15). Thus, **L^OH,OH^** shows two emission mechanisms, i.e., phosphorescence and fluorescence. The photoluminescence quantum yield for **L^H,OH^** and **L^OH,OH^** is less than 1% in the solid state.

Before turning to calculations that will help us identify the emission pathways, it is worthwhile to make a visual inspection of the possible number and nature of the photoluminescence mechanisms by comparing the spectra of **L^H,OH^** and **L^OH,OH^**. As mentioned above, both compounds exhibit phosphorescence with similar lifetimes in the order of one microsecond. Owing to the close wavelength of the maxima of the most intense band (546 nm for **L^H,OH^** and 568 nm for **L^OH,OH^**), we can assume that this band implies the same emission mechanism for both compounds. The shoulder appearing at *ca.* 670 nm in the case of **L^OH,OH^** may be responsible for the short lifetimes and can therefore be attributed to fluorescence. The absence of this shoulder for **L^H,OH^** may indicate that the fluorescence mechanism observed for **L^OH,OH^** cannot be realized for **L^H,OH^**. We hypothesize that this fluorescence mechanism is somehow related to the O^Imid^–H···N^Py^ proton transfer site, which is absent for **L^H,OH^**. 

### 2.6. Elucidation of the Fluorescence and Phosphorescence Mechanisms for **L^H,OH^** and **L^OH,OH^**

**Figure 8:**
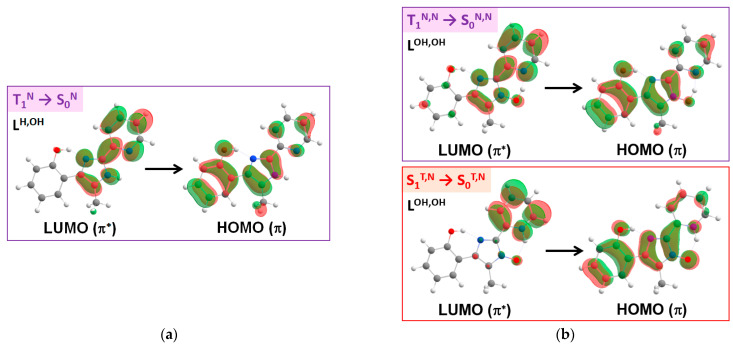
Frontier molecular orbitals related to the emission processes observed for **L^H,OH^** (**a**) and **L^OH,OH^** (**b**).

Geometry optimizations of the excited states were performed in order to establish the photoluminescence mechanisms for **L^H,OH^** and **L^OH,OH^** and to verify our predictions from the previous paragraph. The PEC of the first triplet excited state of **L^H,OH^** reveals two minima, T_1_^N^ and T_1_^T^ (Figure 5). The T_1_^N^ optimized geometry is characterized by a slightly enlarged O^Ph^–H distance (1.006 Å for T_1_^N^ vs. 0.980 Å for S_0_^N^) and a shortened O^Ph^···N^Imid^ hydrogen bond length (2.550 Å for T_1_^N^ vs. 2.627 Å for S_0_^N^) compared with the S_0_^N^ relaxed geometry. The calculated T_1_^N^ → S_0_^N^ phosphorescence wavelength (578 nm) is in excellent agreement with the maximum of the intensive emission band (568 nm). According to the analysis of the frontier molecular orbitals, T_1_^N^ → S_0_^N^ is LUMO → HOMO transition (Figure 8). LUMO is a π*-orbital that is equally located on pyridine and imidazole moieties, whereas HOMO is a π-orbital that is majorly located on hydroxyphenyl and imidazole parts of the molecule. Therefore, the observed T_1_^N^ → S_0_^N^ phosphorescence is associated with charge transfer from the pyridine moiety to the hydroxyphenyl moiety (this is directly opposite to the S_0_^N^ → S_1_^N^ absorption mechanism discussed above). Although the second minimum on the PEC of the T_1_ state, T_1_^T^ (O^Ph^–H 1.841 Å, Figure 5), is thermodynamically more stable than T_1_^N^ by *ca.* 16 kJ/mol, the energy barrier separating T_1_^N^ and T_1_^T^ is as high as *ca.* 14 kJ/mol, which impedes efficient ESIPT in the triplet manifold. Furthermore, the computed T_1_^T^ → S_0_^T^ phosphorescence wavelength (1095 nm) is largely overestimated compared with the position of the phosphorescence band. Thus, we attribute the observed phosphorescence of **L^H,OH^** with τ = 1.05 μs to the T_1_^N^ → S_0_^N^ transition of the *N*-**L^H,OH^** form, which is not related to the ESIPT process.

Having established the phosphorescence mechanism (T_1_^N^ → S_0_^N^) for **L^H,OH^**, the following question arises: how can the molecules of **L^H,OH^** populate the T_1_ state? Classically, in most compounds the triplet manifold is populated after S_0_ → S_1_ excitation followed by S_1_ → T_1_ intersystem crossing. Returning to our discussion of absorption properties, the S_0_^N^ → S_1_^N^ vertical absorption is computed at 336 nm for **L^H,OH^** (Figure 3). At the same time, the phosphorescence band of **L^H,OH^** in the region 450–750 nm is predominantly excited at λ_ex_ = 400–420 nm. Obviously, such low energies cannot lead to the population of the S_1_ state. Therefore, we suggest that in the case of **L^H,OH^** there is a direct population of the triplet manifold from the ground state, S_0_^N^ → T_1_^N^, since only triplets can be populated with λ_ex_ = 400–420 nm (λ_calc. S0-T1_ = 462 nm, λ_calc. S0-T2_ = 395 nm). However, the classical mechanism of populating the T_1_ state (S_0_^N^ → S_1_^N^ → T_1_^N^) is also feasible when molecules are excited with high energy quanta (λ_ex_ < 336 nm). 

**Figure 9:**
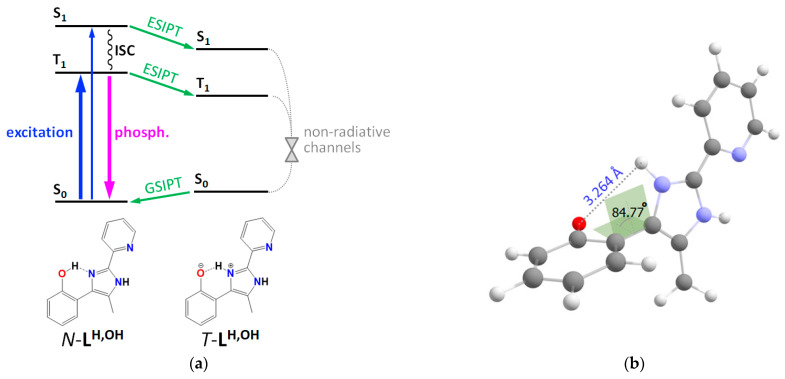
(**a**) Photophysical and photochemical properties of **L^H,OH^** in the solid state summarized in a simplified energy level diagram. ISC—intersystem crossing, phosph.—phosphorescence. (**b**) The geometry of **L^H,OH^** that is close to the conical intersection between the S_0_ and S_1_ states.

In contrast to the triplet manifold, ESIPT is possible for the singlet manifold of **L^H,OH^**. After S_0_^N^ → S_1_^N^ excitation, the ESIPT process is barrierless in the S_1_ state. There are no minima on the PEC of the first singlet excited state, as shown in Figure 5. A non-constrained geometry optimization of the S_1_ state directly leads to a non-planar geometry near the conical intersection (CI) between the S_0_ and S_1_ states (Figure 9b). According to the literature, ESIPT is often coupled with the radiationless deactivation via twisted intramolecular charge transfer (TICT) states of a non-planar biradicaloid nature [83,85,94,95,96,97,98,99]. This non-planarity arises from the twisting around a double-like bond between proton-donating and proton-accepting moieties (around the C^Ph^–C^Imid^ bond in our case). Subsequent ultrafast internal conversion via S_0_/S_1_ CI results in the non-radiative deactivation of the excited twisted phototautomer. Since **L^H,OH^** does not luminesce in solution and weakly luminesces in the solid state, we believe that this non-radiative deactivation is the predominant photophysical process for **L^H,OH^**, which is responsible for emission quenching. It should be noted that the precise geometry of the CI between the S_0_ and S_1_ states can only be optimized using ab initio methods such as CASSCF, CASPT2 or NEVPT2. However, our TDDFT optimization of the S_1_ state leads to the oscillations around the CI geometry, which may serve as an indirect evidence of its existence. Figure 9b shows the geometry at the optimization step closest to the real CI geometry (with the lowest S_0_-S_1_ energy gap of only 2.2 kJ/mol; the dihedral angle between the proton-donating hydroxyphenyl and proton-accepting imidazole moieties reaches 85° at this geometry). 

**L^OH,OH^** has two proton transfer sites and therefore provides more possible emission mechanisms than **L^H,OH^**. The PES of the T_1_ state shows four energy minima: T_1_^N,N^ (O^Ph^–H 1.008 Å, O^Imid^–H 1.065 Å), T_1_^T,N^ (O^Ph^–H 0.994 Å, O^Imid^–H 1.931 Å), T_1_^N,T^ (O^Ph^–H 1.829 Å, O^Imid^–H 1.051 Å) and T_1_^T,T^ (O^Ph^–H 1.807 Å, O^Imid^–H 1.787 Å, Figure 4). The mechanisms of the population of the T_1_ state for **L^OH,OH^** are similar to those for **L^H,OH^**. Upon excitation with high energies (S_0_^N,N^ → S_n_ ^N,N^, where n ≥ 1), the S_1_^N,N^ state can be reached, and the T_1_^N,N^ state can be populated from S_1_^N,N^ via S_1_^N,N^ → T_1_^N,N^ intersystem crossing. Upon excitation with lower energies, the S_1_^N,N^ state cannot be reached, and the T_1_^N,N^ state can be populated only via direct S_0_^N,N^ → T_1_^N,N^ excitation. In comparison with S_0_^N,N^, both hydrogen bonds become stronger in the T_1_^N,N^ energy minimum (O^Ph^–H···N^Imid^: 2.628 Å for S_0_^N,N^ vs. 2.546 Å for T_1_^N,N^; O^Imid^–H···N^Py^: 2.616 Å for S_0_^N,N^ vs. 2.509 Å for T_1_^N,N^). The computed T_1_^N,N^ → S_0_^N,N^ phosphorescence wavelength (586 nm) is in good agreement with the experimental emission maximum (546 nm). It corresponds to LUMO (π*) → HOMO (π) transition of the *N*,*N*-**L^OH,OH^** form, which is not related to ESIPT and has both protons at the oxygen atoms. Same as for **L^H,OH^**, this transition represents charge transfer from the pyridine heterocycle to the hydroxyphenyl moiety (Figure 8).

Three other minima on the T_1_ state PES of **L^OH,OH^**, i.e., T_1_^T,N^, T_1_^N,T^ and T_1_^T,T^, are energetically more favorable than T_1_^N,N^ by *ca.* 58, 14 and 43 kJ/mol, respectively (Figure 4). However, these three minima do not lead to emission for the following reasons. Firstly, the population of the T_1_^N,T^ minimum after S_0_^N,N^ → T_1_^N,N^ excitation is kinetically restricted due to the high energy barrier between the T_1_^N,N^ and T_1_^N,T^ minima (*ca.* 14 kJ/mol). Secondly, although the energy barriers for the T_1_^N,N^ → T_1_^T,N^ and T_1_^N,N^ → T_1_^T,T^ ESIPT processes are significantly lower (*ca.* 1 kJ/mol), the calculated T_1_^T,N^ → S_0_^T,N^ and T_1_^T,T^ → S_0_^T,T^ phosphorescence wavelengths (959 and 1301 nm, respectively) are located in the infrared region and hugely overestimated compared with the experimental phosphorescence band. Owing to the fact that we do not observe luminescence in the infrared region, the molecules that populate the T_1_^T,N^ and T_1_^T,T^ minima most likely deactivate non-radiatively, for example via S_0_/T_1_ conical intersections. Thus, among four possible radiative deactivation channels in the triplet manifold associated with four energy minima, only one (T_1_^N,N^ → S_0_^N,N^) takes place according to the experimental data. 

**Figure 10:**
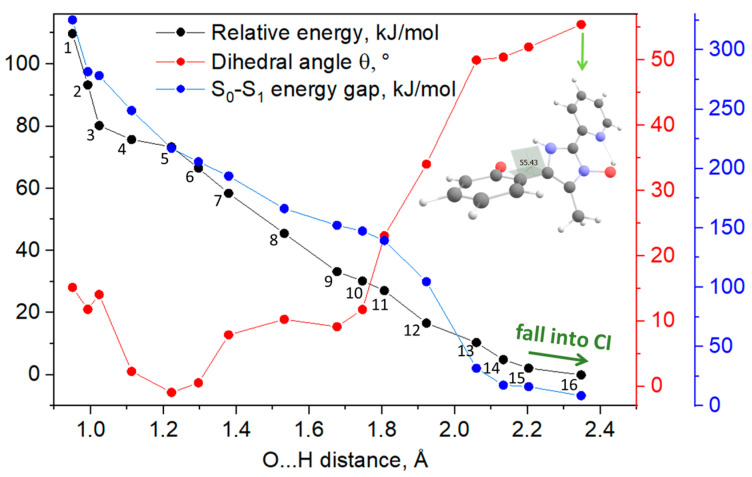
Evolution of energy, dihedral angle θ and the S_0_-S_1_ energy gap during the geometry optimization of the S_1_ state for **L^OH,OH^**. The number of the optimization cycle is shown near the energy curve (black).

We did not plot the PES of the S_1_ state for **L^OH,OH^** because geometry optimizations of the S_1_ state with almost all initial guess structures directly lead to the non-planar near-CI geometry and oscillate around it, proving that most of the molecules that are excited to the S_1_ state deactivate non-radiatively through a conical intersection. A typical evolution of (i) the energy, (ii) dihedral angle θ between the planes of hydroxyphenyl and hydroxyimidazole parts and (iii) the S_0_-S_1_ energy gap during the geometry optimization is shown in Figure 10. Starting from the planar geometry with the O^Ph^–H distance of 0.95 Å, this distance tends to increase during each optimization cycle. In parallel with the energy stabilization, the S_0_-S_1_ energy gap decreases during the optimization process. At the O^Ph^–H distance of 1.75 Å, the dihedral angle θ starts to increase drastically and reaches 55° at the near-CI geometry with the S_0_-S_1_ energy gap of only 7.3 kJ/mol. After the 16th optimization cycle, the optimization process starts oscillating around this near-CI geometry.

**Figure 11:**
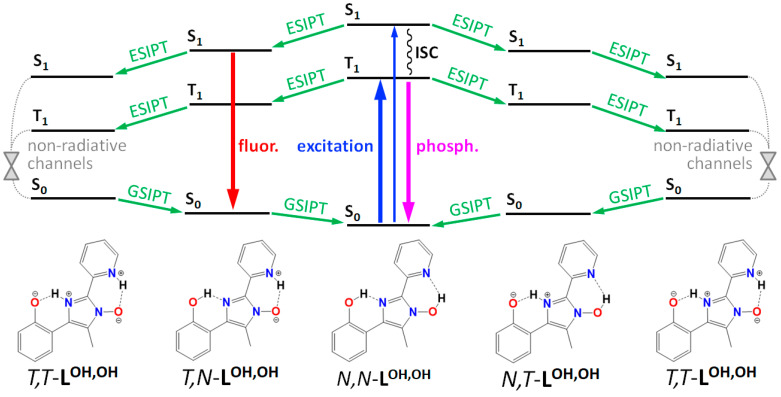
Photophysical and photochemical properties of **L^OH,OH^** in the solid state summarized in a simplified energy level diagram. ISC—intersystem crossing, phosph.—phosphorescence; fluor.—fluorescence.

However, there is one exemption to the above-mentioned trend of radiationless deactivation via CI for **L^OH,OH^**. The geometry of the *T*,*N*-**L^OH,OH^** form can be successfully optimized in the S_1_ state without falling into S_0_/S_1_ CI. The corresponding S_1_^T,N^ → S_0_^T,N^ transition (λ_calc._ = 731 nm, f = 0.0367) is in accordance with the position of the low-energy shoulder in the experimental luminescence spectrum of **L^OH,OH^**. This transition represents charge transfer from the π*-orbital located on pyridine moiety (LUMO) to the π-orbital located on both hydroxyimidazole and hydroxyphenyl moieties (HOMO, Figure 8). Thus, short lifetimes of the excited states observed for **L^OH,OH^** (τ = 2 ns and τ = 21 ns) are due to the S_1_^T,N^ → S_0_^T,N^ fluorescence. Now it becomes obvious that the same low-energy shoulder does not appear for **L^H,OH^** due to the lack of the O^Imid^–H···N^Py^ proton transfer site. Summing up, two major emission channels have been established for **L^OH,OH^**: (i) T_1_^N,N^ → S_0_^N,N^ phosphorescence of the *N*,*N*-**L^OH,OH^** form related to the most intensive emission band at 500–800 nm; and (ii) S_1_^T,N^ → S_0_^T,N^ fluorescence of the *T*,*N*-**L^OH,OH^** form related to the low-energy shoulder at *ca.* 670 nm (Figure 11).

## 3. Materials and Methods

### 3.1. General Information

Elemental analysis was performed with a EuroEA3000 analyzer using standard technique. The IR spectra were recorded in KBr on a Bruker Vector-22 spectrometer.^1^H and ^13^C NMR spectra were recorded on Bruker AV-400 (400.13 and 100.61 MHz) and Bruker DRX-500 (500.13 and 125.76 MHz) spectrometers using the residual signals of the solvent (CDCl_3_) at 7.24 ppm for ^1^H and 76.9 ppm for ^13^C with respect to TMS as the internal standard. Corrected photoluminescence spectra were recorded on a Fluorolog 3 spectrometer (Horiba Jobin Yvon). 

### 3.2. 1-(2-Benzoyloxyphenyl)-2-(Hydroxyimino)Propan-1-One (B)

A solution of isopropyl nitrite (0.74 g, 8.3 mmol) in methanol (5 mL) and then conc. HCl acid (1.4 mL) were added dropwise to a solution of 2-(benzyloxy)propiophenone (**A**) (synthesized according to the procedure reported in ref. [88]) (1.27 g, 5 mmol) in methanol (25 mL) under heating at 40 °C. The reaction mixture was stirred at 40–45 °C for 8 h, cooled and neutralized with a solution of NaHCO_3_. After evaporation to remove methanol, the aqueous layer was extracted with CHCl_3_ and dried over MgSO_4_. After solvent removal under reduced pressure, the residue was purified by column chromatography (silica gel, CHCl_3_) and then triturated with hexane to give the title product. Yield: 0.78 g (55%), m.p. 102–103 °C (100–101 °C [88]). Anal. Calc. for C_16_H_13_NO_4_: C, 67.84; H, 4.62; N, 4.95. Found: C, 67.97; H, 4.62; N, 5.02%. ^1^H NMR (400.13 MHz, CDCl_3_) δ (ppm): 8.30 (s, 1H, OH), 8.08 (d, 2H, *J* = 7.4 Hz, H_Ar_), 7.61 (t, 1H, *J* = 7.4 Hz, H_Ar_), 7.57–7.52 (m, 2H, H_Ar_), 7.47 (m, 2H, H_Ar_), 7.33–7.27 (m, 2H, H_Ar_), 1.97 (s, 3H, Me). ^13^C NMR (125.76 MHz, CDCl_3_) δ (ppm): 189.30, 164.61, 156.71, 148.41, 133.68, 132.04, 130.88, 130.05, 129.98, 128.68, 128.46, 125.53, 122.93, 8.95. IR (KBr, ν cm^−1^): 3311, 1713 (C=O), 1670 (C=O), 1603, 1279, 1269, 1203, 1178, 1115, 1086, 1018, 906, 702, 656. 

### 3.3. 1-Hydroxy-4-(2-Hydroxyphenyl)-5-Methyl-2-(Pyridin-2-yl)-1H-Imidazole (**L^OH,OH^**)

Conc. NH_4_OH (19 mL) and pyridine-2-carboxaldehyde (0.44 g, 4.1 mmol) were added to a solution of 1-(2-benzoyloxyphenyl)-2-(hydroxyimino)propan-1-one (1.13 g, 4 mmol) in a mixture of 1,4-dioxane (16 mL) and EtOH (4 mL). The reaction mixture was stirred at room temperature for 3 days. After removing of the solvent, the residue was purified by column chromatography (silica gel, CHCl_3_) and recrystallized from the hexane-ethylacetate mixture (10:1) to afford the title product. Yield: 0.91 g (85%), m.p. 121–122 °C. Anal. Calc. for C_15_H_13_N_3_O_2_: C, 67.40; H, 4.90; N, 15.72. Found: C, 67.45; H, 5.02; N, 15.81%. ^1^H NMR (400.13 MHz, CDCl_3_) δ (ppm): 13.02 (br. s, 1H, OH), 8.43 (ddd, 1H, *J* = 5.1, 1.5, 0.5 Hz, H_Ar_), 7.99 (dt, 1H, *J* = 7.8, 1.3 Hz, H_Ar_), 7.88 (dt, 1H, *J* = 7.8, 1.3 Hz, H_Ar_), 7.50 (dd, 1H, *J* = 7.8, 1.3 Hz, H_Ar_), 7.28 (ddd, 1H, *J* = 7.5, 5.1, 1.1 Hz, H_Ar_), 7.16 (ddd, 1H, *J* = 8.1, 7.5, 1.5 Hz, H_Ar_), 7.01 (dd, 1H, *J* = 8.1, 1.1 Hz, H_Ar_), 6.88 (dt, 1H, *J* = 7.8, 1.3 Hz, H_Ar_), 2.57 (s, 3H, Me). ^13^C NMR (125.76 MHz, CDCl_3_) δ (ppm): 156.40, 148.37, 145.61, 138.47, 132.56, 128.60, 127.77, 125.42, 122.48, 121.61, 119.42, 118.71, 117.79, 117.08, 9.19. IR (KBr, ν cm^−1^): 1603, 1566, 1489, 1439, 1389, 1288, 1255, 1178, 1153, 1126, 1014, 773, 741, 652. 

### 3.4. 4-(2-Hydroxyphenyl)-5-Methyl-2-(Pyridin-2-yl)-1H-Imidazole (**L^H,OH^**) 

A mixture of 1-hydroxy-4-(2-hydroxyphenyl)-5-methyl-2-(pyridin-2-yl)-1*H*-imidazole (**L^OH,OH^**) (0.19 g, 0.71 mmol), chloroacetone (0.066 g, 0.71 mmol) and dried K_2_CO_3_ (0.11 g, 0.8 mmol) in dried dimethylformamide (6 mL) was stirred at room temperature for 1 h and then at 40–45 °C for 4 h. After cooling the reaction mixture was diluted with water, the residue formed was filtered, washed with water, dried and purified by column chromatography (silica gel, CHCl_3_). The recrystallization of the residue from EtOH afforded **L^H,OH^**. Yield: 0.16 g (89%), m.p. 202–203 °C. Anal. Calc. for C_15_H_13_N_3_O: C, 71.70; H, 5.21; N, 16.72. Found: C, 71.62; H, 5.34; N, 16.65%. ^1^H NMR (400.13 MHz, CDCl_3_) δ (ppm): 12.38 (s, 1H, NH), 11.04 (br. s, 1H, OH), 8.51 (ddd, 1H, *J* = 5.1, 1.3, 0.5 Hz, H_Ar_), 8.07 (dt, 1H, *J* = 8.0, 1.2 Hz, H_Ar_), 7.79 (dt, 1H, *J* = 7.8, 1.2 Hz, H_Ar_), 7.47 (dd, 1H, *J* = 7.8, 1.2 Hz, H_Ar_), 7.25 (ddd, 1H, *J* = 7.5, 5.1, 1.2 Hz, H_Ar_), 7.17 (ddd, 1H, *J* = 8.0, 7.5, 1.4 Hz, H_Ar_), 7.03 (dd, 1H, *J* = 7.8, 1.4 Hz, H_Ar_), 6.88 (dt, 1H, *J* = 7.5, 1.3 Hz, H_Ar_), 2.53 (s, 3H, Me). ^13^C NMR (100.61 MHz, CDCl_3_) δ (ppm): 156.38, 148.64, 147.49, 141.49, 137.62, 136.87, 127.97, 125.68, 124.42, 123.37, 120.15, 118.91, 118.30, 117.25, 12.47. IR (KBr, ν cm^−1^): 3311, 1597, 1578, 1443, 1400, 1286, 1244, 1134, 999, 825, 783, 756, 742, 700.

### 3.5. X-ray Crystallography

Diffraction data for single-crystal **L^OH,OH^** were obtained at 291 K on an automated four-circle Agilent Xcalibur diffractometer equipped with an area AtlasS2 detector (graphite monochromator, λ(MoKα) = 0.71073 Å, ω-scans with a step 0.25°). Integration, absorption correction, and determination of unit cell parameters were performed using the CrysAlisPro program package [100]. The structure was solved by dual space algorithm (SHELXT [101]) and refined by the full-matrix least squares technique (SHELXL [102]) in the anisotropic approximation (except hydrogen atoms). Positions of hydrogen atoms were calculated geometrically and refined in the riding model. The crystallographic data and details of the structure refinements are summarized in Supplementary Materials (Table S1). CCDC 2237906 contains the supplementary crystallographic data for this paper. These data can be obtained free of charge from The Cambridge Crystallographic Data Center at http://www.ccdc.cam.ac.uk/structures/ (accessed on 26 January 2023).

### 3.6. Computational Details

The quantum chemical calculations presented in this study were conducted using density functional theory (DFT), time-dependent DFT (TDDFT) and Tamm–Dancoff approximated DFT (TDADFT) methods in Gaussian 16 software package [103]. We used the hybrid exchange-correlation functional PBE0 [104] since our previous studies demonstrated its satisfying performance in modeling photophysical and photochemical properties of organic ESIPT-emitters [83,85]. Compared with probably the best known hybrid functional B3LYP, PBE0 provides absorption energies that are closer to the experimental data, while B3LYP tends to red-shift some vertical absorptions for **L^H,OH^** and **L^OH,OH^** (Supplementary Materials, Figures S12 and S13). The 6–31 + G(d) basis set was used for all atoms [105,106,107,108,109]. Absorption spectra were calculated on ground state geometries using TDDFT. Singlet excited state geometries (S_1_) as well as S_1_-S_0_ fluorescence energies were also determined using the TDDFT approach. The optimizations of the lowest triplet excited state (T_1_) geometries of **L^H,OH^** and **L^OH,OH^** were carried out by an unrestricted DFT (uDFT) method. Subsequent single-point TDADFT computations on T_1_ optimized geometries revealed T_1_-S_0_ phosphorescence energies. The use of TDADFT rather than TDDFT in the latter case is justified by the fact that the Tamm–Dancoff approximation tends to strongly correct the computed triplet state energies comparatively to TDDFT. Relaxed T_1_ state geometries can also be obtained using TDDFT or TDADFT approaches; however, the uDFT method is more preferable because it requires much less computational cost. In the case of absorption spectra, the solvent effects of acetonitrile molecules were considered by the polarizable continuum model (PCM), and all other computations were performed in the gas phase. The D3 version of Grimme’s dispersion with Becke–Johnson damping was employed for each calculation. Potential energy curves (PECs) and surfaces (PESes) of the desired states (S_0_, S_1_, T_1_) along the proton transfer reaction were plotted by scanning the O...H bond distance between 0.95 and 2.00 Å with a step of 0.05 Å. All frequencies in the harmonic approximation for the calculated global minimum energy geometries were positive, confirming that the optimized molecular geometries correspond to the real minima on the potential energy surfaces. The atomic coordinates of all optimized geometries are given in Supplementary Materials (Tables S3–S16). The geometries and molecular orbitals were visualized using ChemCraft software [110].

## 4. Conclusions

In this work we presented the synthesis of imidazole-based ESIPT-capable compounds, 1-hydroxy-4-(2-hydroxyphenyl)-5-methyl-2-(pyridin-2-yl)-1*H*-imidazole (**L^OH,OH^**) and 4-(2-hydroxyphenyl)-5-methyl-2-(pyridin-2-yl)-1*H*-imidazole (**L^H,OH^**). In the **L^OH,OH^** trinuclear molecule, the central moiety, i.e., the 1-hydroxy-1*H*-imidazole one, is decorated with the proton-donating and proton-accepting peripheral groups and, therefore, under photoexcitation can act both as a proton acceptor and a proton donor in the ESIPT reactions. Importantly, we found a convenient synthetic pathway for the conversion of 1-hydroxy-4-(2-hydroxyphenyl)-1*H*-imidazoles to 4-(2-hydroxyphenyl)-1*H*-imidazoles. This synthetic pathway is based on the reaction of the 1-hydroxy-4-(2-hydroxyphenyl)-1*H*-imidazole derivative with chloroacetone. Despite chloroacetone being known to interact with phenolic hydroxy groups, in our case the reaction proceeded selectively with the imidazolic hydroxy group only, leaving the phenolic hydroxy group unaffected. Thus, this reaction has high synthetic potential for selective reduction of 1-hydroxy-1*H*-imidazoles decorated with hydroxyphenyl groups to corresponding 1*H*-imidazoles. 

A slight structural difference between these two compounds leads to significant changes in their photoluminescence response. **L^H,OH^** emits in the light green region, while **L^OH,OH^** luminesces in the orange region. According to our computations, both emitters share the same emission mechanism, i.e., phosphorescence of the normal form of the molecule (T_1_^N^ → S_0_^N^ for the *N-***L^H,OH^** form and T_1_^N,N^ → S_0_^N,N^ for the *N*,*N-***L^OH,OH^** form), which is not related to ESIPT. After the ESIPT process, both compounds can decay non-radiatively through S_0_/S_1_ and S_0_/T_1_ conical intersections, which explains their low photoluminescence quantum yield. The phosphorescence band is the most intensive for both compounds. However, **L^OH,OH^** also exhibits fluorescence of the *T*,*N-***L^OH,OH^** form, S_1_^T,N^ → S_0_^T,N^, with one proton transferred from the hydroxyimidazole moiety to the pyridine moiety. This fluorescence mechanism is responsible for the appearance of the low-energy shoulder in the emission spectrum of **L^OH,OH^**. Thus, owing to the presence of two proton transfer sites, **L^OH,OH^** appears to be a rare example of ESIPT-emitters that exhibit fluorescence and phosphorescence simultaneously.

## Figures and Tables

**Table 1:** The most relevant geometric parameters for the optimized ground and excited state geometries of **L^H,OH^** and **L^OH,OH^**.

Cmpd.	State	O^Ph^–H, Å	O^Ph^–H···N^Imid^, Å	O^Imid^–H, Å	O^Imid^–H···N^Py^, Å	θ_1_,° ^a^	θ_2_,° ^b^
**L^H,OH^**	S_0_^N^	0.980	2.627	–	–	14.97	0.38
	T_1_^N^	1.006	2.550	–	–	5.79	0.20
	T_1_^T^	1.841	2.603	–	–	4.14	0.25
	near-CI ^c^	3.264	3.411	–	–	84.77	1.92
**L^H,OH^**	S_0_^N,N^	0.988	2.628	1.010	2.616	14.78	0.92
	S_0_^T,N^	0.992	2.592	1.595	2.542	0.79	0.04
	S_1_^T,N^	0.998	2.584	1.785	2.674	0.00	0.23
	T_1_^N,N^	1.008	2.546	1.065	2.509	−0.02	0.00
	T_1_^N,T^	1.829	2.599	1.051	2.531	−0.01	0.00
	T_1_^T,N^	0.994	2.590	1.931	2.728	−0.02	0.00
	T_1_^T,T^	1.807	2.593	1.787	2.648	−0.02	−0.01
	near-CI ^c^	2.347	2.922	0.964	2.732	55.43	5.45

^a^—θ_1_ is the dihedral angle between the planes of hydroxyphenyl and imidazole moieties. ^b^—θ_2_ is the dihedral angle between the planes of pyridine and imidazole moieties. ^c^—geometries that are close to the conical intersection between the S_0_ and S_1_ states.

## Data Availability

Data will be made available on request.

## Supplementary Materials

The following supporting information can be downloaded at: https://www.mdpi.com/article/10.3390/molecules28041793/s1, Tables S1–S16 and Figures S1–S15: characterization data and quantum chemical calculations data.

## Sample Availability

Samples of the compounds are available from the authors.
