# Supplementary material for: ESIPT-Capable 4-(2-Hydroxyphenyl)-2-(Pyridin-2-yl)-1H-Imidazoles with Single and Double Proton Transfer: Synthesis, Selective Reduction of the Imidazolic OH Group and Luminescence
Source: Molecules. 2023 Feb 14;28(4):1793. doi: 10.3390/molecules28041793 (PMC9962989; doi:10.3390/molecules28041793)
Supplement: Supplementary file 1 [file molecules-28-01793-s001.zip › molecules-2213293-supplementary.pdf]

Electronic Supplementary Information

ESIPT-capable 4-(2-hydroxyphenyl)-2-(pyridin-2-yl)-1*H*-imidazoles with single and double proton transfer: synthesis, selective reduction of the imidazolic OH group and luminescence

Nikita A. Shekhovtsov [1,*], Elena B. Nikolaenkova [2], Alexey A. Ryadun [1], Denis G. Samsonenko [1], Alexsei Ya. Tikhonov [2,*] Mark B. Bushuev [1,*]

[1] Nikolaev Institute of Inorganic Chemistry, Siberian Branch of Russian Academy of Sciences, 3, Acad. Lavrentiev Ave., Novosibirsk, 630090, Russia.
[2] N. N. Vorozhtsov Novosibirsk Institute of Organic Chemistry, Siberian Branch of Russian Academy of Sciences, 9, Acad. Lavrentiev Ave., Novosibirsk, 630090, Russia.
Correspondence: shekhovtsov@niic.nsc.ru (Nikita A. Shekhovtsov), alyatikh@nioch.nsc.ru (Alexsei Ya. Tikhonov), bushuev@niic.nsc.ru (Mark B. Bushuev).

Table of Contents

Spectral data 3
- Figure S1 3
- Figure S2 3
- Figure S3 4
- Figure S4 5
- Figure S5 5
- Figure S6 6
- Figure S7 7
- Figure S8 7

Structural data 8
- Table S1 8
- Table S2 9
- Figure S9 12
- Figure S10 12
- Figure S11 12

Photophysical data 13
- Figure S12 13
- Figure S13 13
- Figure S14 14
- Figure S15 15

Theoretical data 16
- Table S3 16
- Table S4 17
- Table S5 18
- Table S6 19
- Table S7 20
- Table S8 21
- Table S9 22
- Table S10 23
- Table S11 24
- Table S12 25
- Table S13 26
- Table S14 27
- Table S15 28
- Table S16 29

Spectral data

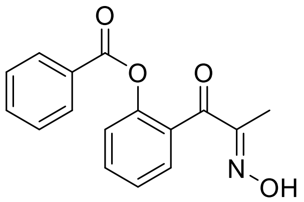

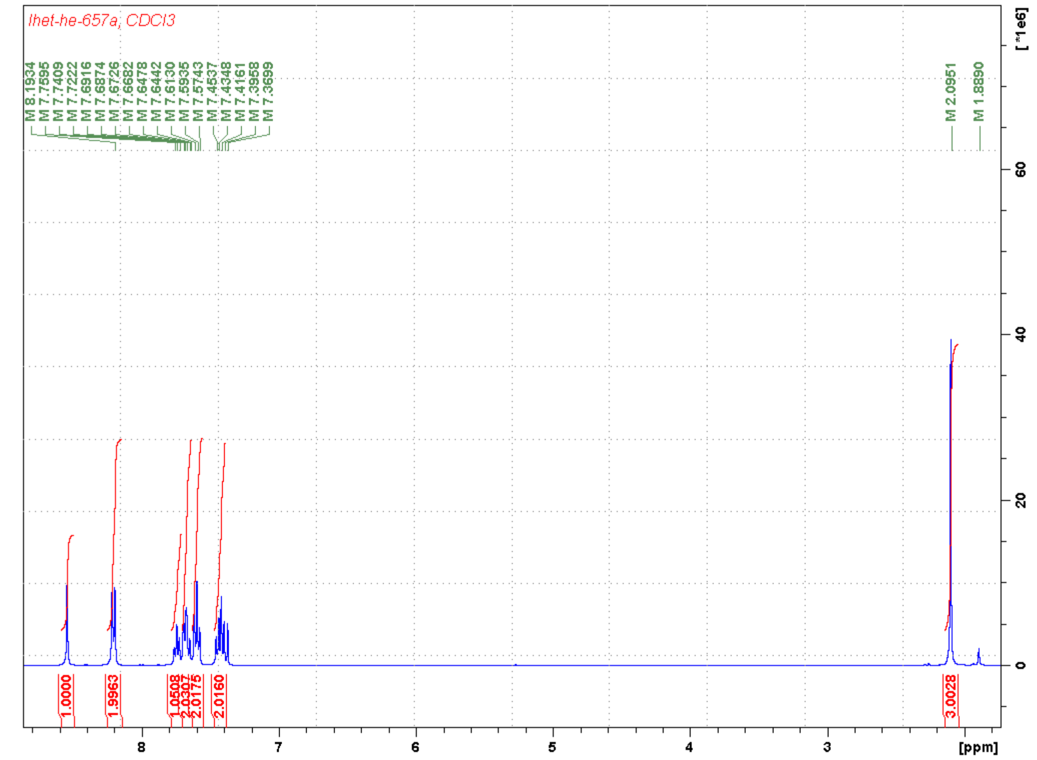

Figure S1. ^{1}H NMR spectrum of 1-(2-benzoyloxyphenyl)-2-(hydroxyimino)propan-1-one (in $CDCl_3$).

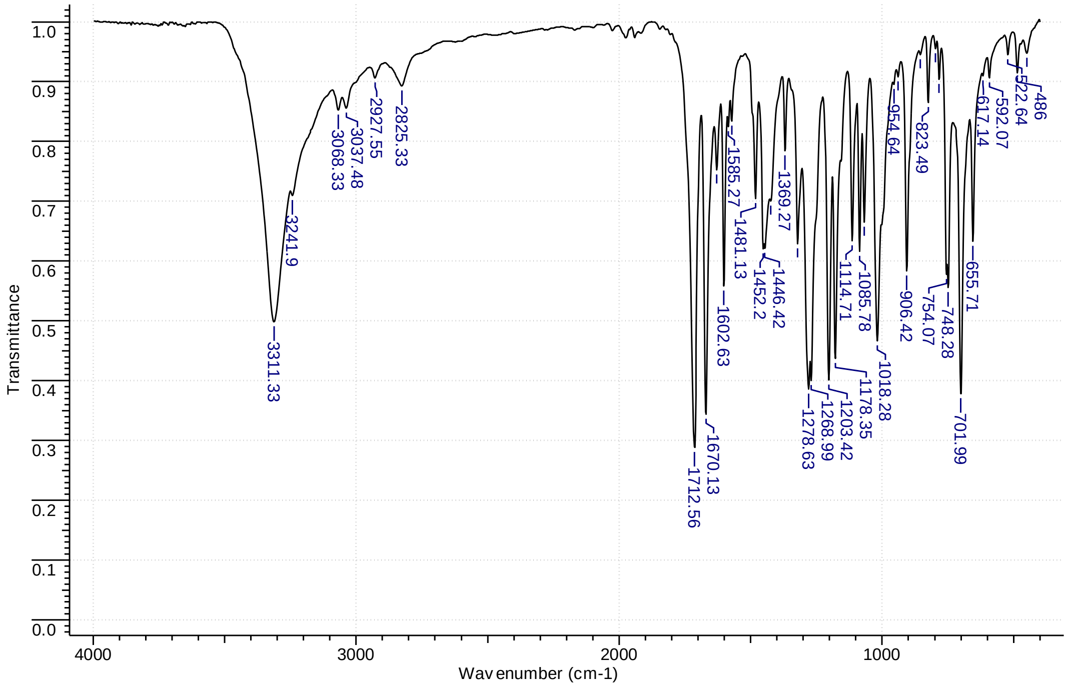

Figure S2. IR spectrum of 1-(2-benzoyloxyphenyl)-2-(hydroxyimino)propan-1-one (in KBr).

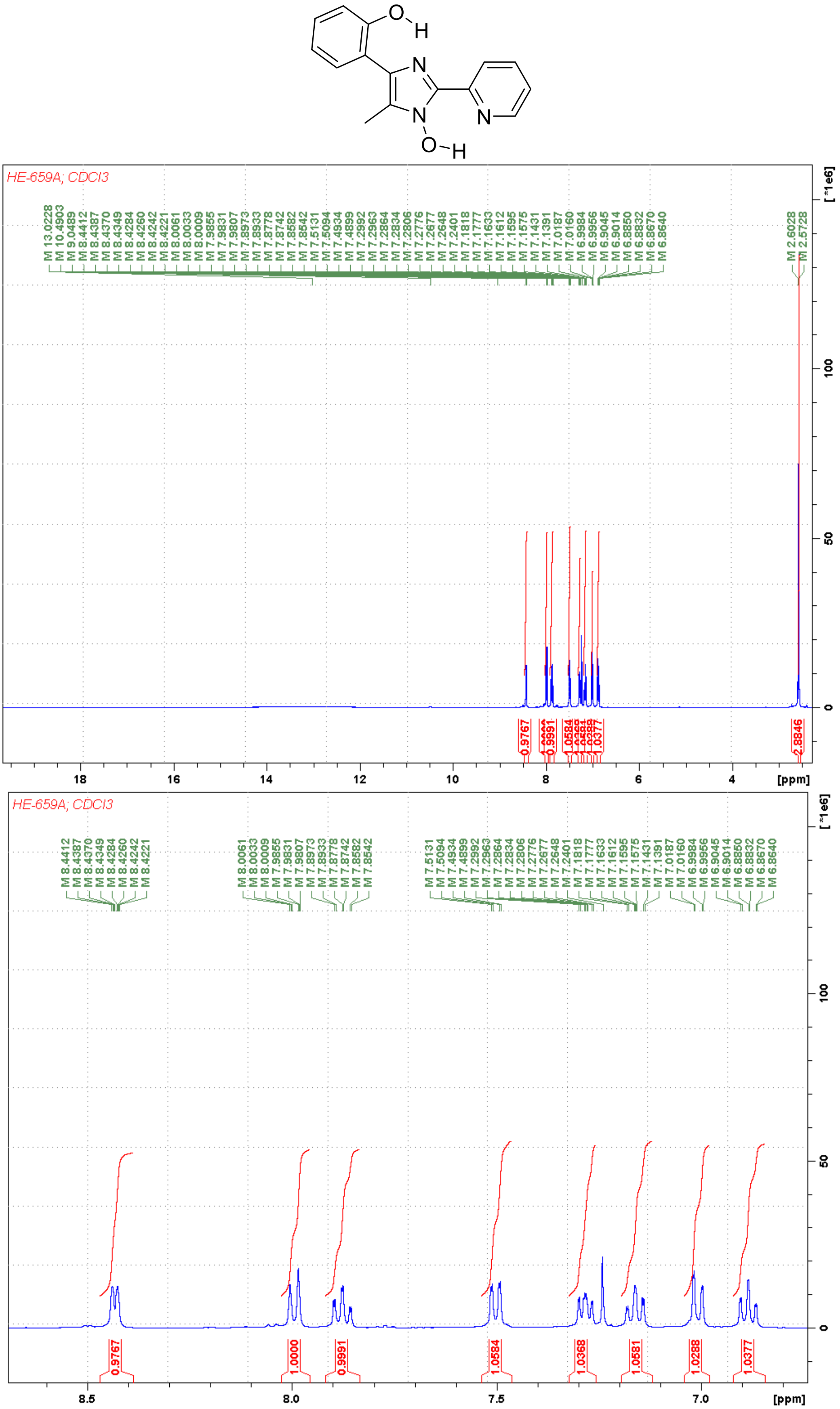

Figure S3. ^{1}H NMR spectrum of 1-hydroxy-4-(2-hydroxyphenyl)-5-methyl-2-(pyridin-2-yl)-1*H*-imidazole (in $CDCl_3$).

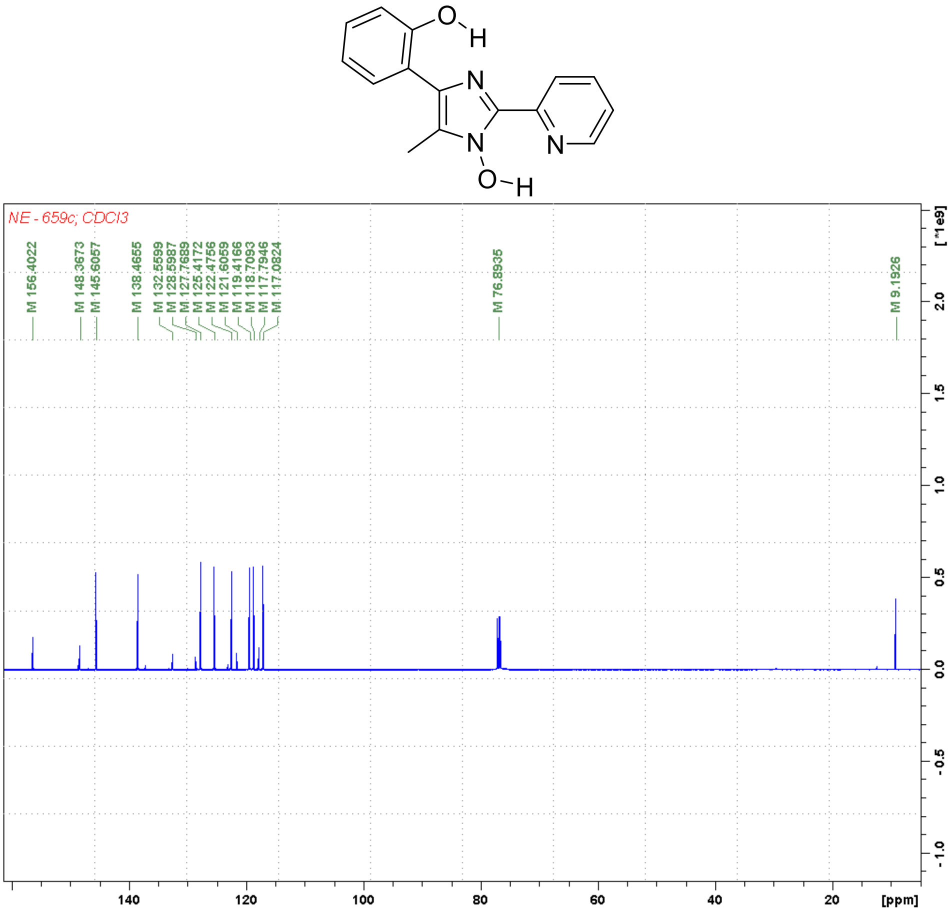

Figure S4. ^{13}C NMR spectrum of 1-hydroxy-4-(2-hydroxyphenyl)-5-methyl-2-(pyridin-2-yl)-1*H*-imidazole (in $CDCl_3$).

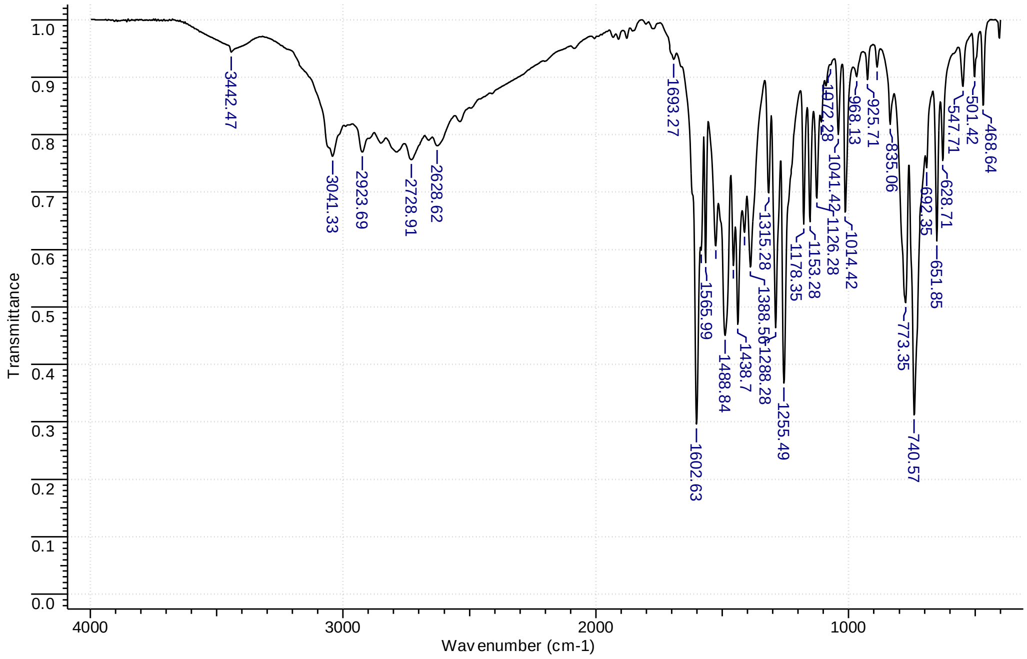

Figure S5. IR spectrum of 1-hydroxy-4-(2-hydroxyphenyl)-5-methyl-2-(pyridin-2-yl)-1*H*-imidazole (in KBr).

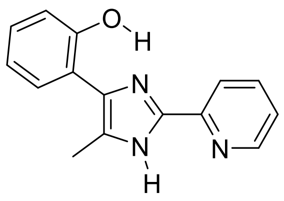

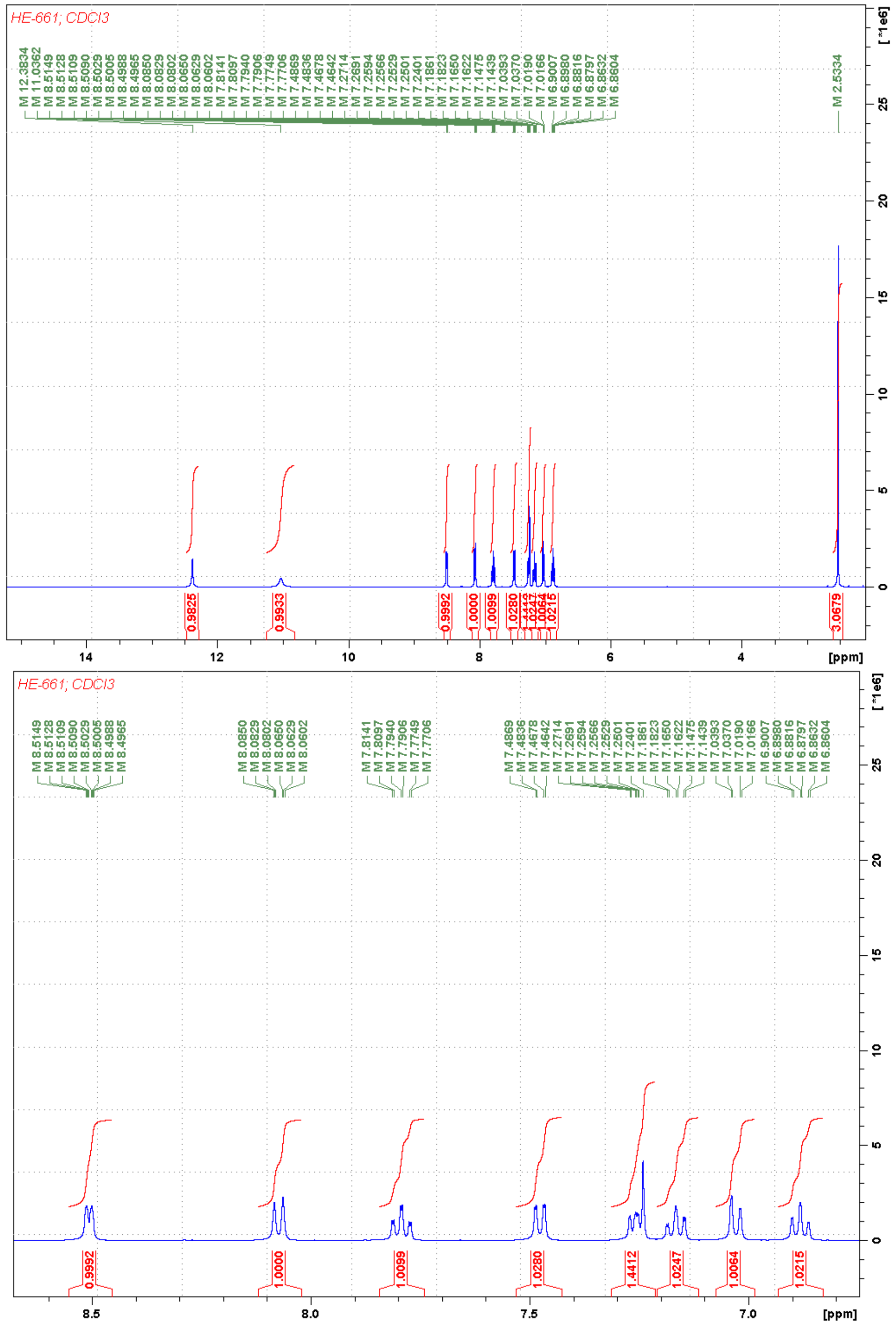

Figure S6. ^{1}H NMR spectrum of 4-(2-hydroxyphenyl)-5-methyl-2-(pyridin-2-yl)-1*H*-imidazole (in $CDCl_3$).

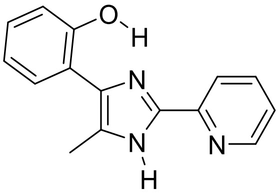

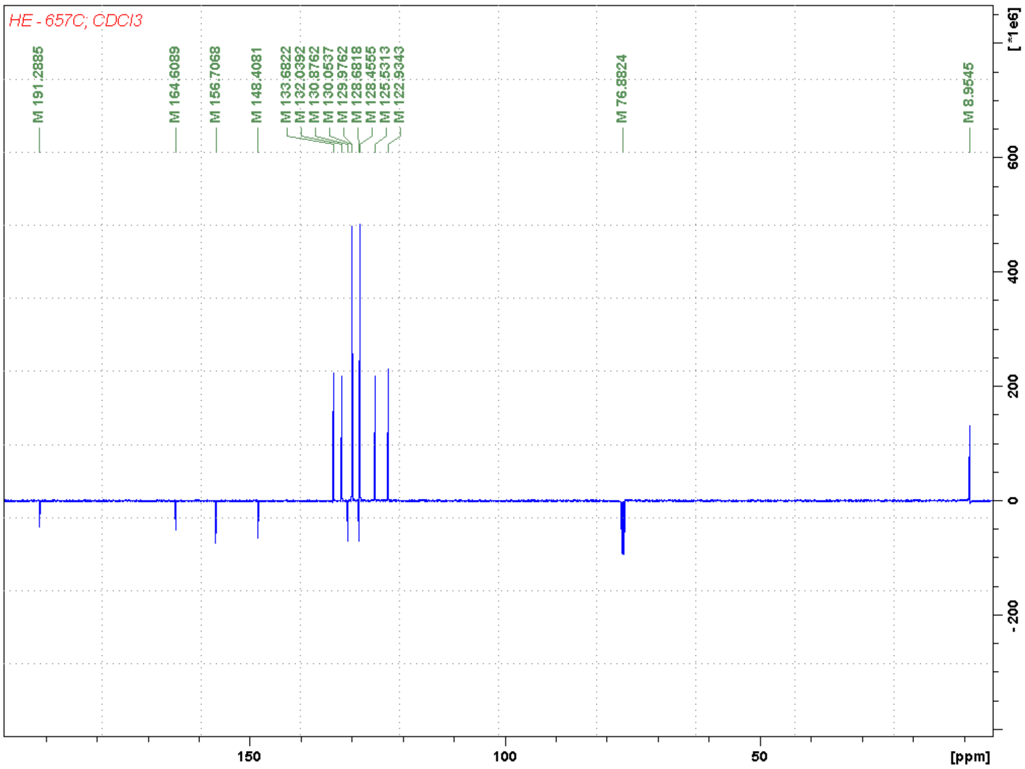

Figure S7. ^{13}C NMR spectrum of 1-(2-benzoyloxyphenyl)-2-(hydroxyimino)propan-1-one (in $CDCl_3$).

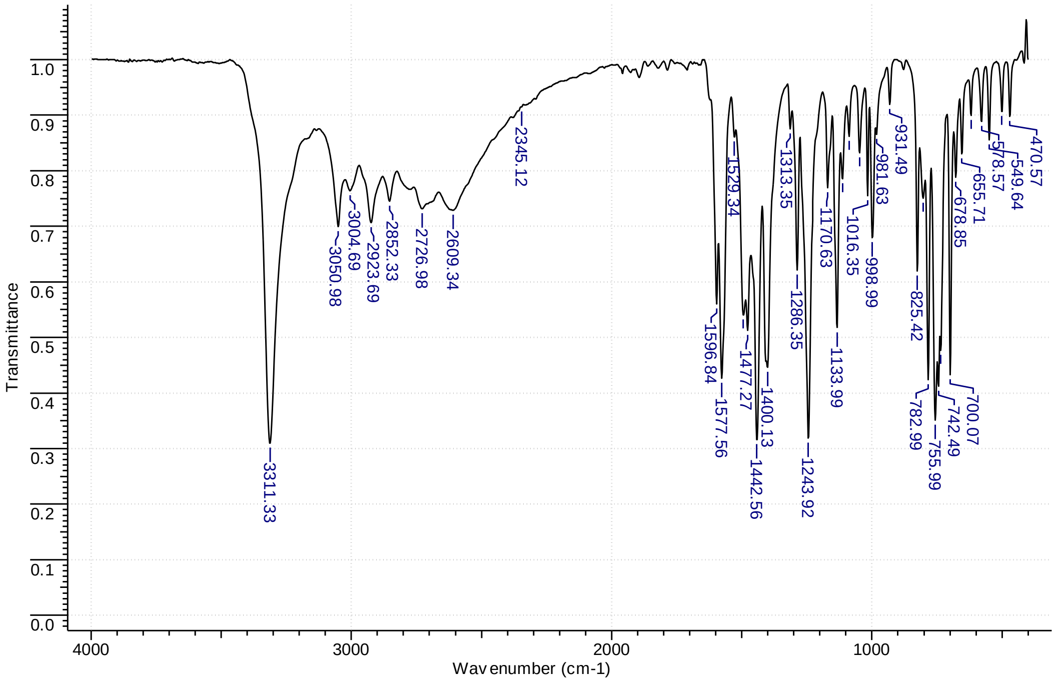

Figure S8. IR spectrum of 4-(2-hydroxyphenyl)-5-methyl-2-(pyridin-2-yl)-1*H*-imidazole (in KBr).

Structural data

Table S1. Crystal data and structure refinement for **L^OH,OH^**.

Identification code	**L^OH,OH^**
Empirical formula	$C_{15}H_{13}N_3O_2$
Formula weight	267.28
Crystal system	*Monoclinic*
Space group	$P2_1/c$
a, Å	13.0513(5)
b, Å	7.2703(4)
c, Å	27.5672(12)
β, deg.	90.491(4)
V, Å^3	2615.7(2)
Z	8
D(calc.), g/cm^3	1.357
μ, mm^{-1}	0.093
F(000)	1120
Crystal size, mm	0.47 × 0.11 × 0.05
θ range for data collection, deg.	2.14–29.13
Index ranges	$-16 \le h \le 17$, $-9 \le k \le 9$, $-25 \le l \le 36$
Reflections collected / independent	13699 / 5878
R_{int}	0.0195
Reflections with $I > 2\sigma(I)$	3781
Goodness-of-fit on F^2	1.040
Final R indices [$I > 2\sigma(I)$]	$R_1 = 0.0507$, $wR_2 = 0.1191$
R indices (all data)	$R_1 = 0.0875$, $wR_2 = 0.1332$
Largest diff. peak / hole, e/Å^3	0.172 / −0.178

Table S2. Bond lengths and angles for $\mathbf{L}^{OH,OH}$.

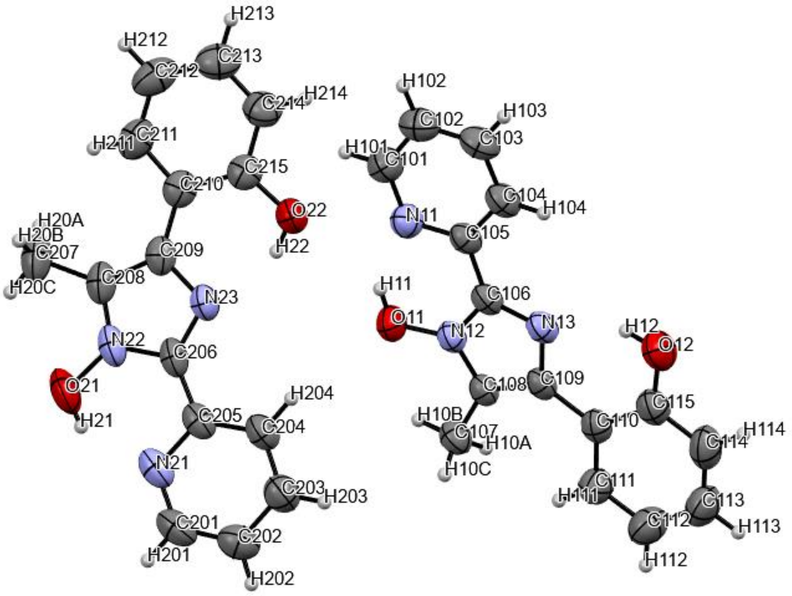

Bond	*d*, Å	Bond	*d*, Å
O(11)-H(11)	0.8200	O(21)-H(21)	0.8200
O(11)-N(12)	1.3826(16)	O(21)-N(22)	1.3832(18)
O(12)-H(12)	0.8200	O(22)-H(22)	0.8200
O(12)-C(115)	1.355(2)	O(22)-C(215)	1.3560(18)
N(11)-C(101)	1.336(2)	N(21)-C(201)	1.339(2)
N(11)-C(105)	1.3475(19)	N(21)-C(205)	1.349(2)
N(12)-C(106)	1.3582(19)	N(22)-C(206)	1.353(2)
N(12)-C(108)	1.3556(19)	N(22)-C(208)	1.353(2)
N(13)-C(106)	1.3241(19)	N(23)-C(206)	1.330(2)
N(13)-C(109)	1.3802(19)	N(23)-C(209)	1.376(2)
C(101)-H(101)	0.9300	C(201)-H(201)	0.9300
C(101)-C(102)	1.369(2)	C(201)-C(202)	1.370(3)
C(102)-H(102)	0.9300	C(202)-H(202)	0.9300
C(102)-C(103)	1.384(2)	C(202)-C(203)	1.376(3)
C(103)-H(103)	0.9300	C(203)-H(203)	0.9300
C(103)-C(104)	1.372(2)	C(203)-C(204)	1.374(2)
C(104)-H(104)	0.9300	C(204)-H(204)	0.9300
C(104)-C(105)	1.388(2)	C(204)-C(205)	1.388(2)
C(105)-C(106)	1.452(2)	C(205)-C(206)	1.455(2)
C(107)-H(10A)	0.9600	C(207)-H(20A)	0.9600
C(107)-H(10B)	0.9600	C(207)-H(20B)	0.9600

C(107)-H(10C)	0.9600	C(207)-H(20C)	0.9600
C(107)-C(108)	1.485(2)	C(207)-C(208)	1.490(2)
C(108)-C(109)	1.393(2)	C(208)-C(209)	1.393(2)
C(109)-C(110)	1.464(2)	C(209)-C(210)	1.466(2)
C(110)-C(111)	1.389(2)	C(210)-C(211)	1.396(2)
C(110)-C(115)	1.407(2)	C(210)-C(215)	1.412(2)
C(111)-H(111)	0.9300	C(211)-H(211)	0.9300
C(111)-C(112)	1.380(2)	C(211)-C(212)	1.376(3)
C(112)-H(112)	0.9300	C(212)-H(212)	0.9300
C(112)-C(113)	1.368(3)	C(212)-C(213)	1.375(3)
C(113)-H(113)	0.9300	C(213)-H(213)	0.9300
C(113)-C(114)	1.367(3)	C(213)-C(214)	1.370(3)
C(114)-H(114)	0.9300	C(214)-H(214)	0.9300
C(114)-C(115)	1.399(3)	C(214)-C(215)	1.385(2)
Angle	ω, deg.	Angle	ω, deg.
N(12)-O(11)-H(11)	109.5	N(22)-O(21)-H(21)	109.5
C(115)-O(12)-H(12)	109.5	C(215)-O(22)-H(22)	109.5
C(101)-N(11)-C(105)	117.87(14)	C(201)-N(21)-C(205)	118.01(16)
C(106)-N(12)-O(11)	126.15(13)	C(206)-N(22)-O(21)	125.28(15)
C(108)-N(12)-O(11)	123.03(12)	C(208)-N(22)-O(21)	123.53(14)
C(108)-N(12)-C(106)	110.80(13)	C(208)-N(22)-C(206)	111.19(14)
C(106)-N(13)-C(109)	107.10(13)	C(206)-N(23)-C(209)	106.83(13)
N(11)-C(101)-H(101)	118.4	N(21)-C(201)-H(201)	118.5
N(11)-C(101)-C(102)	123.24(17)	N(21)-C(201)-C(202)	122.94(17)
C(102)-C(101)-H(101)	118.4	C(202)-C(201)-H(201)	118.5
C(101)-C(102)-H(102)	120.7	C(201)-C(202)-H(202)	120.6
C(101)-C(102)-C(103)	118.64(16)	C(201)-C(202)-C(203)	118.72(18)
C(103)-C(102)-H(102)	120.7	C(203)-C(202)-H(202)	120.6
C(102)-C(103)-H(103)	120.3	C(202)-C(203)-H(203)	120.1
C(104)-C(103)-C(102)	119.31(16)	C(204)-C(203)-C(202)	119.74(18)
C(104)-C(103)-H(103)	120.3	C(204)-C(203)-H(203)	120.1
C(103)-C(104)-H(104)	120.6	C(203)-C(204)-H(204)	120.8
C(103)-C(104)-C(105)	118.74(16)	C(203)-C(204)-C(205)	118.46(16)
C(105)-C(104)-H(104)	120.6	C(205)-C(204)-H(204)	120.8
N(11)-C(105)-C(104)	122.20(14)	N(21)-C(205)-C(204)	122.12(16)
N(11)-C(105)-C(106)	115.43(13)	N(21)-C(205)-C(206)	115.43(15)
C(104)-C(105)-C(106)	122.37(15)	C(204)-C(205)-C(206)	122.44(14)
N(12)-C(106)-C(105)	123.33(14)	N(22)-C(206)-C(205)	123.20(14)

N(13)-C(106)-N(12)	108.69(13)	N(23)-C(206)-N(22)	108.54(15)
N(13)-C(106)-C(105)	127.97(14)	N(23)-C(206)-C(205)	128.24(14)
H(10A)-C(107)-H(10B)	109.5	H(20A)-C(207)-H(20B)	109.5
H(10A)-C(107)-H(10C)	109.5	H(20A)-C(207)-H(20C)	109.5
H(10B)-C(107)-H(10C)	109.5	H(20B)-C(207)-H(20C)	109.5
C(108)-C(107)-H(10A)	109.5	C(208)-C(207)-H(20A)	109.5
C(108)-C(107)-H(10B)	109.5	C(208)-C(207)-H(20B)	109.5
C(108)-C(107)-H(10C)	109.5	C(208)-C(207)-H(20C)	109.5
N(12)-C(108)-C(107)	120.69(13)	N(22)-C(208)-C(207)	120.98(16)
N(12)-C(108)-C(109)	103.96(13)	N(22)-C(208)-C(209)	103.70(14)
C(109)-C(108)-C(107)	135.32(14)	C(209)-C(208)-C(207)	135.31(18)
N(13)-C(109)-C(108)	109.45(13)	N(23)-C(209)-C(208)	109.74(15)
N(13)-C(109)-C(110)	120.08(14)	N(23)-C(209)-C(210)	120.26(14)
C(108)-C(109)-C(110)	130.47(14)	C(208)-C(209)-C(210)	130.00(15)
C(111)-C(110)-C(109)	122.45(15)	C(211)-C(210)-C(209)	122.72(17)
C(111)-C(110)-C(115)	116.60(15)	C(211)-C(210)-C(215)	116.64(16)
C(115)-C(110)-C(109)	120.95(15)	C(215)-C(210)-C(209)	120.63(14)
C(110)-C(111)-H(111)	118.6	C(210)-C(211)-H(211)	118.9
C(112)-C(111)-C(110)	122.79(18)	C(212)-C(211)-C(210)	122.3(2)
C(112)-C(111)-H(111)	118.6	C(212)-C(211)-H(211)	118.9
C(111)-C(112)-H(112)	120.2	C(211)-C(212)-H(212)	120.0
C(113)-C(112)-C(111)	119.6(2)	C(213)-C(212)-C(211)	120.03(18)
C(113)-C(112)-H(112)	120.2	C(213)-C(212)-H(212)	120.0
C(112)-C(113)-H(113)	120.0	C(212)-C(213)-H(213)	120.3
C(114)-C(113)-C(112)	120.01(18)	C(214)-C(213)-C(212)	119.34(19)
C(114)-C(113)-H(113)	120.0	C(214)-C(213)-H(213)	120.3
C(113)-C(114)-H(114)	119.6	C(213)-C(214)-H(214)	119.3
C(113)-C(114)-C(115)	120.80(19)	C(213)-C(214)-C(215)	121.42(19)
C(115)-C(114)-H(114)	119.6	C(215)-C(214)-H(214)	119.3
O(12)-C(115)-C(110)	122.19(16)	O(22)-C(215)-C(210)	122.54(15)
O(12)-C(115)-C(114)	117.59(17)	O(22)-C(215)-C(214)	117.30(16)
C(114)-C(115)-C(110)	120.21(18)	C(214)-C(215)-C(210)	120.16(16)

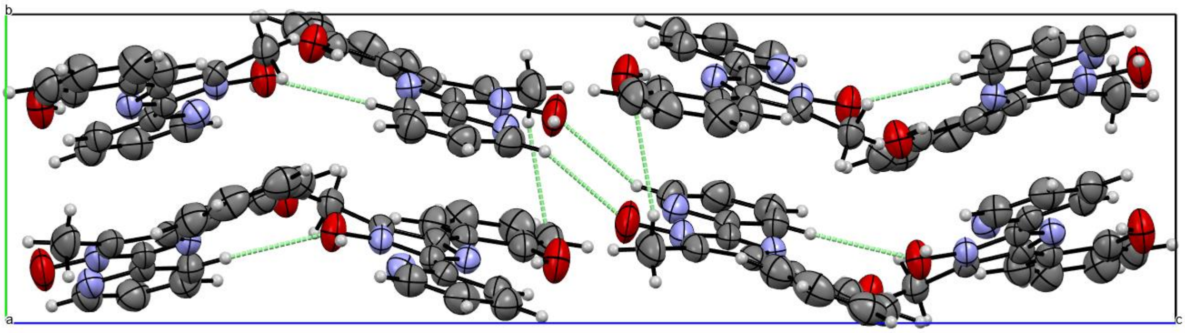

Figure S9. Packing of **L**$^{\mathbf{OH,OH}}$ (view along the *a* axis).

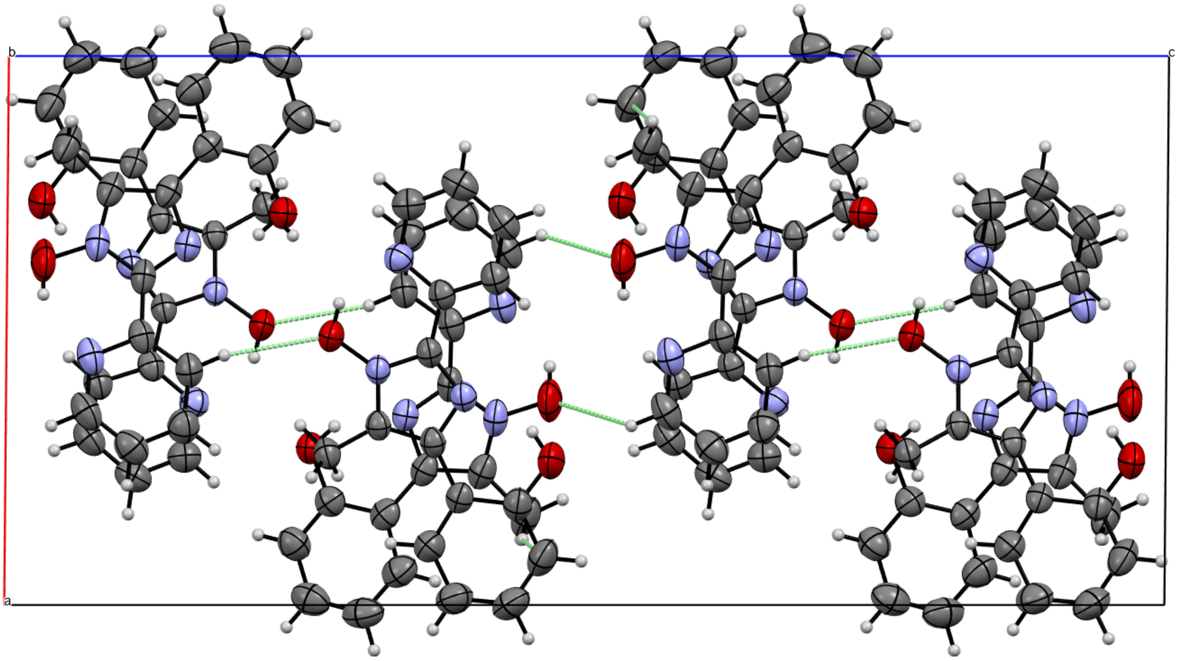

Figure S10. Packing of **L**$^{\mathbf{OH,OH}}$ (view along the *b* axis).

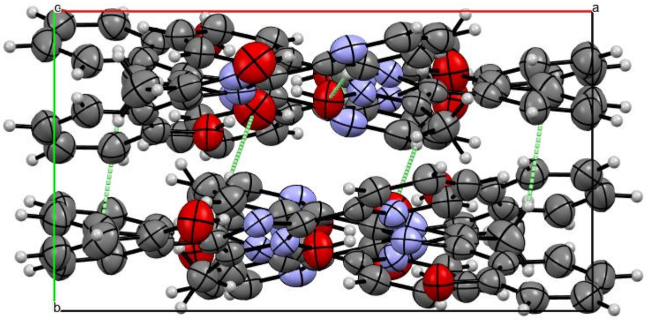

Figure S11. Packing of **L**$^{\mathbf{OH,OH}}$ (view along the *c* axis).

Photophysical data

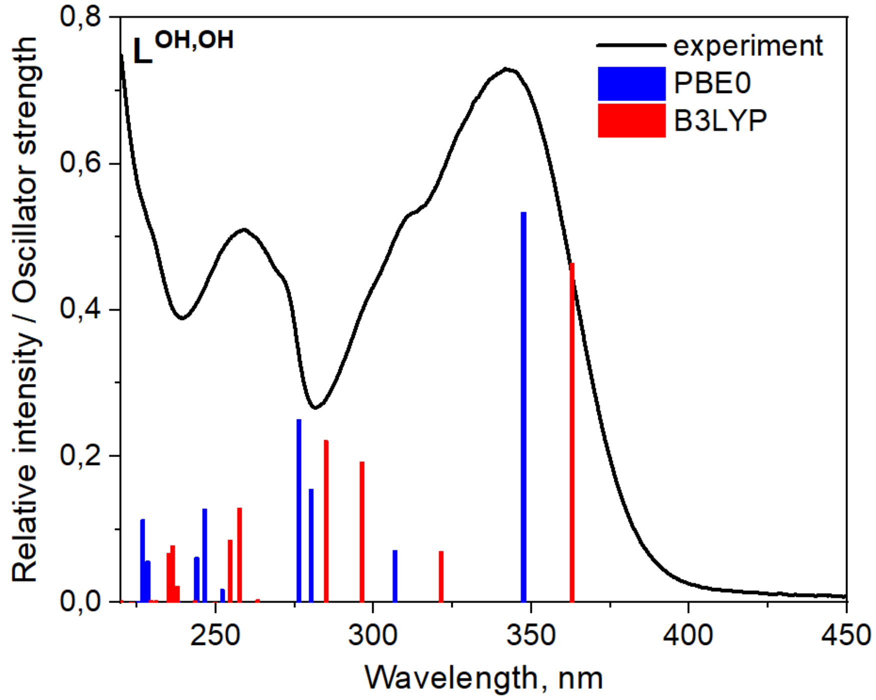

Figure S12. Comparison of the experimental absorption spectrum of $\mathbf{L}^{\mathbf{OH,OH}}$ with those computed using PBE0 and B3LYP functionals.

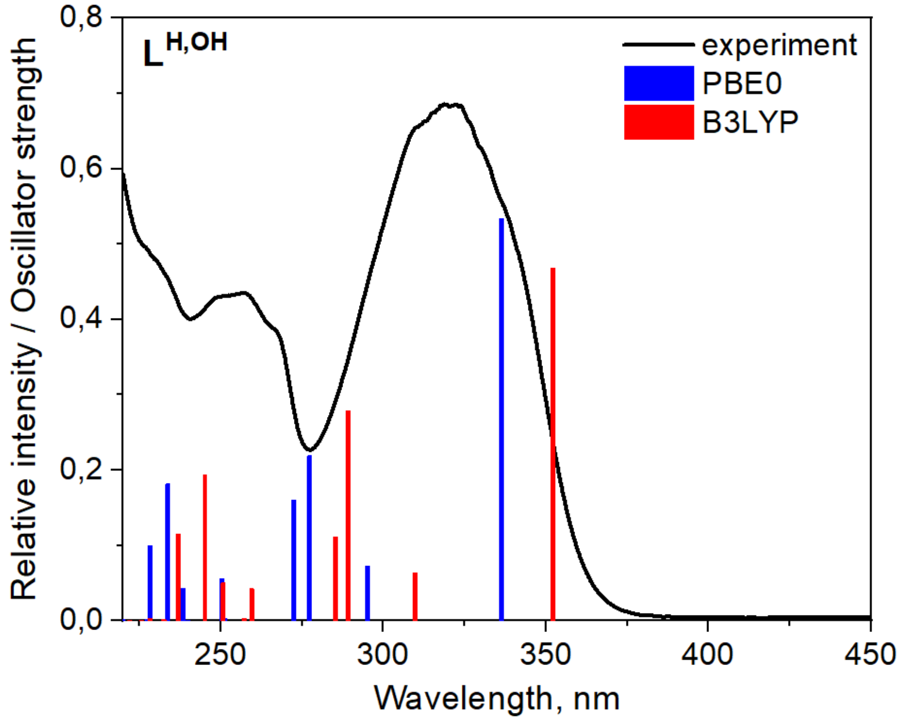

Figure S13. Comparison of the experimental absorption spectrum of $\mathbf{L}^{\mathbf{H,OH}}$ with those computed using PBE0 and B3LYP functionals.

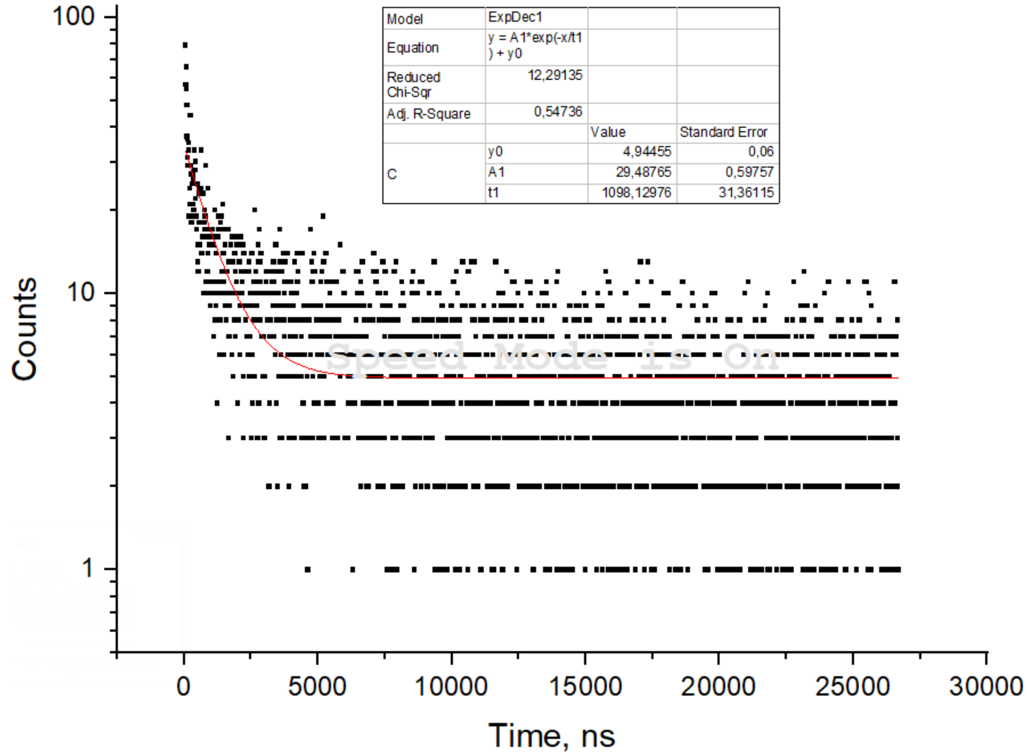

Figure S14. Photoluminescence decay curve for $\mathbf{L}^{H,OH}$.

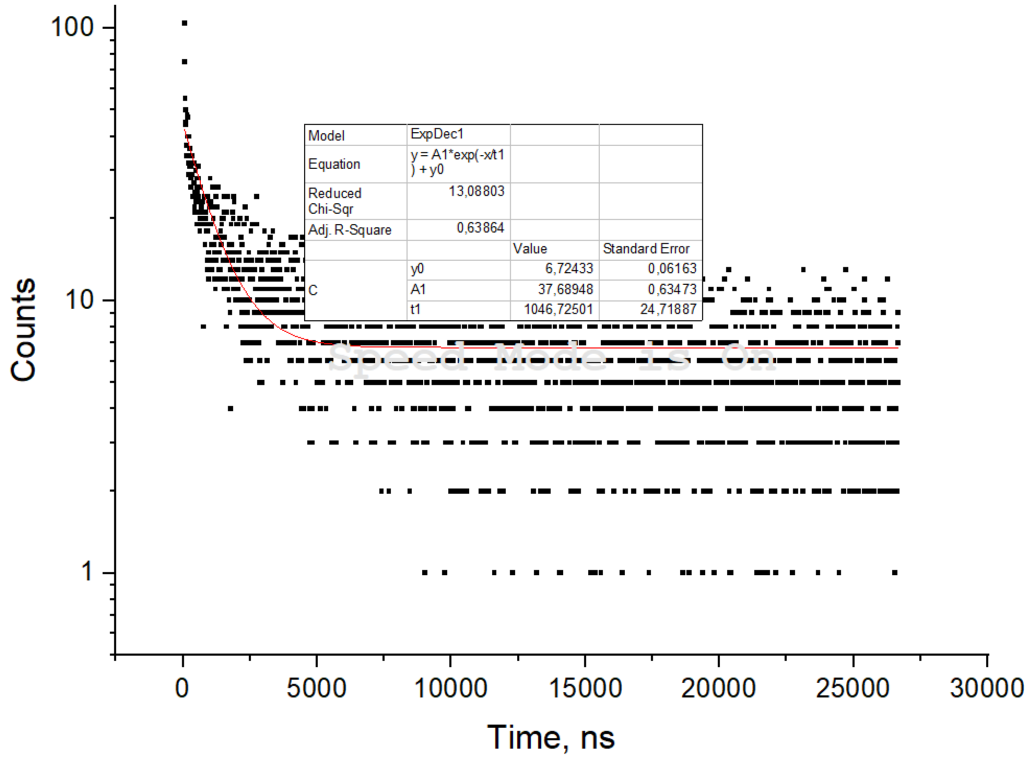

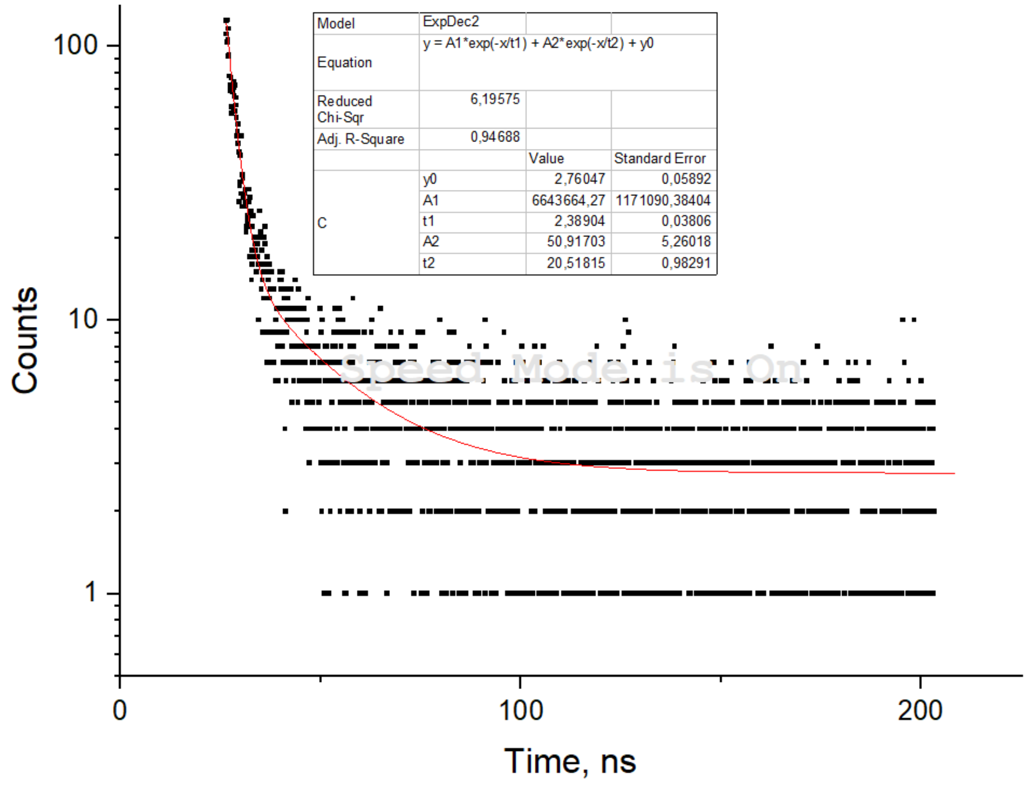

Figure S15. Photoluminescence decay curves for $\mathbf{L}^{OH,OH}$.

Theoretical data

Table S3. Optimized geometry of the **S_0 state** of **$L^{H,OH}$** (***N*-$L^{H,OH}$ form**, **S_0^N**) in Cartesian (XYZ) coordinates as calculated in Gaussian at the PBE0/6-31+g(d) level of theory **in MeCN continuum solvation model.**

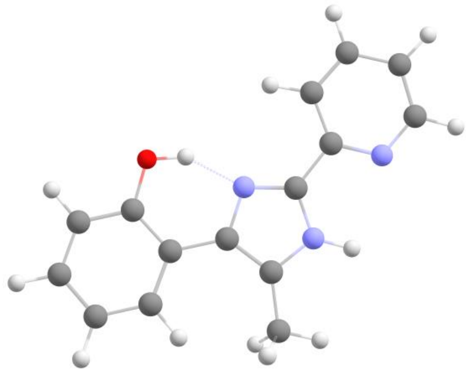

N	0.154602000000	-0.327532000000	-0.028976000000
C	1.253141000000	0.405811000000	-0.006470000000
C	-0.915083000000	0.542549000000	-0.008188000000
H	-3.245774000000	1.944987000000	-0.190102000000
C	-0.434023000000	1.850876000000	0.032257000000
N	0.926463000000	1.718995000000	0.028119000000
H	1.609212000000	2.466225000000	0.053500000000
C	2.633622000000	-0.059486000000	-0.014297000000
H	4.547823000000	-2.851404000000	-0.084168000000
N	3.571391000000	0.901203000000	0.016135000000
C	2.938141000000	-1.423891000000	-0.051148000000
C	4.849301000000	0.521111000000	0.010809000000
C	4.274952000000	-1.800236000000	-0.055962000000
C	5.256380000000	-0.811286000000	-0.024336000000
H	2.140521000000	-2.159136000000	-0.075614000000
H	5.585437000000	1.322031000000	0.035892000000
H	6.312595000000	-1.061331000000	-0.026896000000
C	-1.081068000000	3.189899000000	0.082401000000
H	-1.629570000000	3.411173000000	-0.840505000000
H	-1.787067000000	3.261683000000	0.916733000000
C	-3.399414000000	0.874878000000	-0.111578000000
H	-0.329912000000	3.973619000000	0.215761000000
C	-2.284190000000	0.024979000000	-0.022201000000
C	-2.528103000000	-1.370332000000	0.054486000000
C	-3.838991000000	-1.856201000000	0.057355000000
C	-4.701113000000	0.387806000000	-0.110816000000
C	-4.920399000000	-0.987815000000	-0.022611000000
H	-3.985186000000	-2.931279000000	0.120969000000
H	-5.536758000000	1.078240000000	-0.181759000000
H	-5.932016000000	-1.385382000000	-0.020359000000
O	-1.533485000000	-2.282397000000	0.129114000000
H	-0.667542000000	-1.790889000000	0.078495000000

Table S4. Optimized geometry of the **S_0 state** of **$L^{H,OH}$** (***N*-$L^{H,OH}$ form**, **S_0^N**) in Cartesian (XYZ) coordinates as calculated in Gaussian at the PBE0/6-31+g(d) level of theory **in the gas phase**.

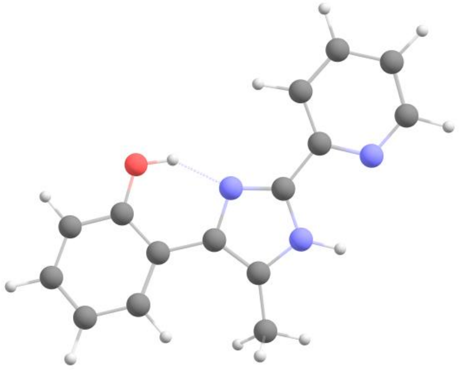

N	-1.142467000000	0.307306000000	-3.002752000000
C	-0.095718000000	1.078464000000	-3.228272000000
C	-0.660009000000	-0.916686000000	-2.594297000000
H	-0.130111000000	-3.196620000000	-1.213189000000
C	0.732962000000	-0.877168000000	-2.586888000000
N	1.047133000000	0.395175000000	-2.980663000000
H	1.966744000000	0.799897000000	-3.097824000000
C	-0.083507000000	2.461675000000	-3.678776000000
H	-2.090108000000	5.044062000000	-4.551231000000
N	1.132364000000	3.007783000000	-3.838765000000
C	-1.272680000000	3.156700000000	-3.922139000000
C	1.193839000000	4.272333000000	-4.251182000000
C	-1.187071000000	4.473174000000	-4.352051000000
C	0.070225000000	5.049944000000	-4.522851000000
H	-2.226429000000	2.662108000000	-3.770229000000
H	2.193972000000	4.684960000000	-4.370786000000
H	0.181613000000	6.076845000000	-4.857264000000
C	1.787827000000	-1.883834000000	-2.291704000000
H	1.935040000000	-2.028778000000	-1.213863000000
H	1.531700000000	-2.855532000000	-2.725903000000
C	-1.151391000000	-3.150704000000	-1.576822000000
H	2.748323000000	-1.573264000000	-2.716665000000
C	-1.576949000000	-2.010943000000	-2.277034000000
C	-2.934975000000	-1.948447000000	-2.676772000000
C	-3.789486000000	-3.023466000000	-2.414214000000
C	-2.005755000000	-4.212178000000	-1.310536000000
C	-3.331214000000	-4.147608000000	-1.742721000000
H	-4.820659000000	-2.942552000000	-2.745830000000
H	-1.643179000000	-5.077716000000	-0.763388000000
H	-4.013252000000	-4.970498000000	-1.544268000000
O	-3.461319000000	-0.887219000000	-3.316239000000
H	-2.775260000000	-0.175472000000	-3.342017000000

Table S5. Optimized geometry of the $\mathbf{T_1}$ **state** of $\mathbf{L^{H,OH}}$ (***N***-$\mathbf{L^{H,OH}}$ **form**, $\mathbf{T_1^N}$) in Cartesian (XYZ) coordinates as calculated in Gaussian at the PBE0/6-31+g(d) level of theory **in the gas phase**.

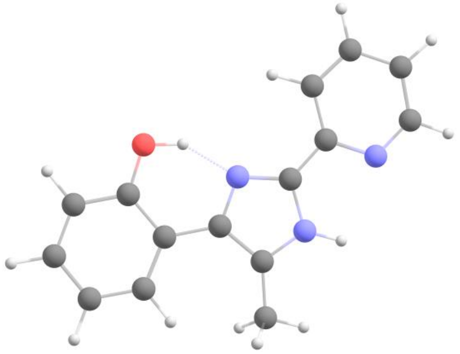

N	0.121619000000	-0.075677000000	-4.090932000000
C	0.159237000000	1.067937000000	-3.334976000000
C	-0.041392000000	-1.109837000000	-3.244828000000
H	-0.617868000000	-3.401353000000	-1.867887000000
C	-0.102466000000	-0.639018000000	-1.888385000000
N	0.006758000000	0.712238000000	-1.986757000000
H	0.039149000000	1.405617000000	-1.252261000000
C	0.316888000000	2.382376000000	-3.722694000000
H	0.740463000000	4.435919000000	-6.415873000000
N	0.315942000000	3.307876000000	-2.678945000000
C	0.471860000000	2.777280000000	-5.096195000000
C	0.464097000000	4.571545000000	-3.010104000000
C	0.621965000000	4.106060000000	-5.387231000000
C	0.620978000000	5.047624000000	-4.327900000000
H	0.464501000000	2.016553000000	-5.870761000000
H	0.461875000000	5.282053000000	-2.182688000000
H	0.737408000000	6.110337000000	-4.513446000000
C	-0.231482000000	-1.312738000000	-0.573799000000
H	-1.231071000000	-1.744147000000	-0.429109000000
H	0.498831000000	-2.121753000000	-0.460770000000
C	-0.406574000000	-3.566353000000	-2.917531000000
H	-0.062714000000	-0.596468000000	0.236040000000
C	-0.110172000000	-2.463111000000	-3.745481000000
C	0.120324000000	-2.709846000000	-5.133157000000
C	0.080246000000	-4.025323000000	-5.619607000000
C	-0.451478000000	-4.855238000000	-3.411253000000
C	-0.199812000000	-5.081040000000	-4.772857000000
H	0.268450000000	-4.178005000000	-6.677932000000
H	-0.685559000000	-5.684346000000	-2.750359000000
H	-0.230990000000	-6.092515000000	-5.169633000000
O	0.380361000000	-1.740483000000	-6.004857000000
H	0.341290000000	-0.864759000000	-5.510884000000

Table S6. Optimized geometry of the $\mathbf{T_1}$ **state** of $\mathbf{L^{H,OH}}$ (***T*-$\mathbf{L^{H,OH}}$ form**, $\mathbf{T_1^T}$) in Cartesian (XYZ) coordinates as calculated in Gaussian at the PBE0/6-31+g(d) level of theory **in the gas phase**.

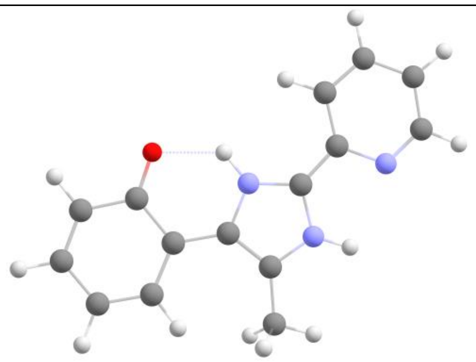

N	-0.104130000000	-0.077890000000	-4.090866000000
C	-0.045145000000	1.085772000000	-3.378701000000
C	-0.051182000000	-1.177881000000	-3.242785000000
H	-0.152065000000	-3.490643000000	-1.812554000000
C	0.035913000000	-0.674144000000	-1.943495000000
N	0.027142000000	0.689167000000	-2.050695000000
H	0.097541000000	1.382365000000	-1.318361000000
C	-0.050015000000	2.421273000000	-3.771174000000
H	-0.181794000000	4.545819000000	-6.427138000000
N	0.028705000000	3.318084000000	-2.724940000000
C	-0.127849000000	2.847286000000	-5.131339000000
C	0.030262000000	4.604662000000	-3.032574000000
C	-0.123134000000	4.194586000000	-5.400000000000
C	-0.042063000000	5.115238000000	-4.334165000000
H	-0.190676000000	2.117986000000	-5.935152000000
H	0.093613000000	5.289333000000	-2.186404000000
H	-0.035708000000	6.186356000000	-4.507088000000
C	0.132565000000	-1.329396000000	-0.614164000000
H	-0.787977000000	-1.867095000000	-0.355514000000
H	0.961885000000	-2.044335000000	-0.576260000000
C	-0.120418000000	-3.634474000000	-2.885846000000
H	0.305835000000	-0.579259000000	0.163050000000
C	-0.081319000000	-2.513629000000	-3.741813000000
C	-0.078369000000	-2.746784000000	-5.197658000000
C	-0.087713000000	-4.118785000000	-5.651276000000
C	-0.133208000000	-4.923313000000	-3.377712000000
C	-0.112827000000	-5.166012000000	-4.775191000000
H	-0.078982000000	-4.264719000000	-6.727353000000
H	-0.166206000000	-5.760270000000	-2.685903000000
H	-0.122509000000	-6.189231000000	-5.142102000000
O	-0.066338000000	-1.809323000000	-6.033846000000
H	-0.105602000000	-0.224226000000	-5.098116000000

Table S7. Optimized geometry of $\mathbf{L^{H,OH}}$ near S_0/S_1 conical intersection in Cartesian (XYZ) coordinates as calculated in Gaussian at the PBE0/6-31+g(d) level of theory **in the gas phase**.

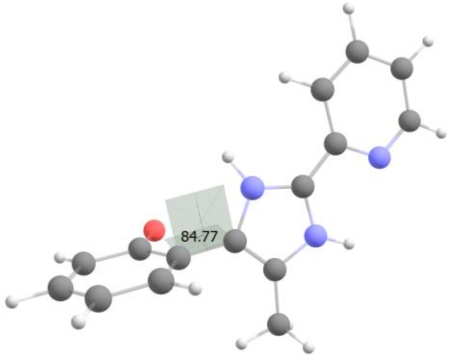

N	-0.305971000000	-0.037272000000	-4.019431000000
C	-0.071671000000	1.114771000000	-3.314699000000
C	0.076517000000	-1.120526000000	-3.219580000000
H	-1.986216000000	-2.800214000000	-2.880325000000
C	0.459618000000	-0.655260000000	-2.021128000000
N	0.352022000000	0.729339000000	-2.082550000000
H	0.573797000000	1.403536000000	-1.364503000000
C	-0.242016000000	2.457670000000	-3.703776000000
H	-1.176723000000	4.526003000000	-6.232906000000
N	0.100622000000	3.367200000000	-2.731070000000
C	-0.714414000000	2.853897000000	-4.985635000000
C	-0.009404000000	4.647960000000	-3.041397000000
C	-0.814457000000	4.196688000000	-5.262723000000
C	-0.450552000000	5.136600000000	-4.277076000000
H	-1.001135000000	2.116144000000	-5.731113000000
H	0.269113000000	5.345396000000	-2.251263000000
H	-0.512119000000	6.203868000000	-4.459404000000
C	0.908637000000	-1.351594000000	-0.793244000000
H	0.216408000000	-1.198009000000	0.044593000000
H	0.974015000000	-2.424661000000	-0.981300000000
C	-1.182407000000	-3.243766000000	-3.465413000000
H	1.900819000000	-0.998287000000	-0.484289000000
C	-0.053145000000	-2.510757000000	-3.705078000000
C	1.010747000000	-3.071693000000	-4.514602000000
C	0.839161000000	-4.414461000000	-5.015558000000
C	-1.315189000000	-4.563139000000	-3.957979000000
C	-0.296498000000	-5.139781000000	-4.725438000000
H	1.659559000000	-4.828934000000	-5.594156000000
H	-2.221612000000	-5.123349000000	-3.753289000000
H	-0.410266000000	-6.155281000000	-5.094919000000
O	2.031076000000	-2.401948000000	-4.782716000000
H	-0.220109000000	-0.049955000000	-5.013214000000

Table S8. Optimized geometry of the **S_0 state** of **$L^{OH,OH}$** (***N,N*-$L^{H,OH}$ form**, **$S_0^{N,N}$**) in Cartesian (XYZ) coordinates as calculated in Gaussian at the PBE0/6-31+g(d) level of theory **in MeCN continuum solvation model**.

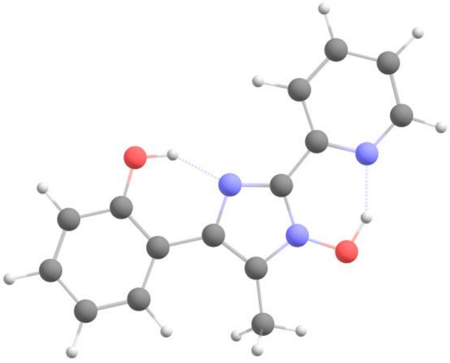

N	-0.170318000000	-0.049413000000	-4.064017000000
C	-0.084930000000	1.054424000000	-3.331537000000
C	-0.066182000000	-1.115928000000	-3.211206000000
C	0.098176000000	-0.652883000000	-1.900436000000
C	0.304502000000	-1.301095000000	-0.580403000000
N	0.071327000000	0.701741000000	-2.027446000000
C	-0.136888000000	2.431619000000	-3.780158000000
N	-0.009692000000	3.363480000000	-2.815837000000
C	-0.306413000000	2.768949000000	-5.126816000000
C	-0.046101000000	4.653554000000	-3.154393000000
C	-0.341765000000	4.113502000000	-5.466565000000
C	-0.209651000000	5.079320000000	-4.467172000000
H	-0.407013000000	1.987814000000	-5.872654000000
H	0.060664000000	5.364747000000	-2.339723000000
H	-0.471746000000	4.409520000000	-6.503515000000
H	-0.232924000000	6.138987000000	-4.698454000000
H	-0.623311000000	-1.734233000000	-0.187884000000
H	1.047379000000	-2.101524000000	-0.651051000000
H	0.662105000000	-0.566034000000	0.144878000000
C	-0.112358000000	-2.483649000000	-3.727076000000
C	-0.019986000000	-2.728746000000	-5.120395000000
C	-0.247175000000	-3.593468000000	-2.876878000000
C	-0.027819000000	-4.039458000000	-5.605193000000
C	-0.260436000000	-4.895487000000	-3.363048000000
C	-0.142795000000	-5.117616000000	-4.736095000000
H	-0.358967000000	-3.432982000000	-1.810221000000
H	0.052415000000	-4.188968000000	-6.678673000000
H	-0.367580000000	-5.729031000000	-2.674812000000
O	0.080924000000	-1.733308000000	-6.029553000000
H	-0.004159000000	-0.869329000000	-5.544356000000
O	0.193620000000	1.554501000000	-0.975316000000
H	0.153327000000	2.463401000000	-1.434090000000
H	-0.150331000000	-6.129724000000	-5.132292000000

Table S9. Optimized geometry of the **S_0 state** of $L^{OH,OH}$ (***N,N*-$L^{H,OH}$ form**, $S_0^{N,N}$) in Cartesian (XYZ) coordinates as calculated in Gaussian at the PBE0/6-31+g(d) level of theory **in the gas phase**.

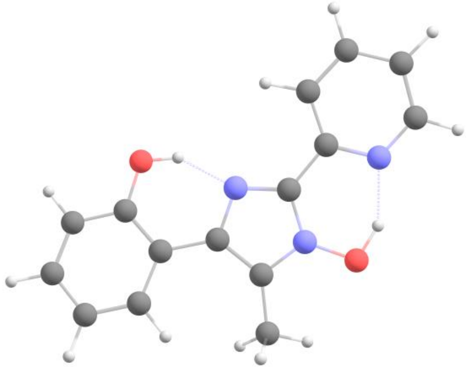

N	-0.187803000000	-0.038195000000	-4.061322000000
C	-0.091463000000	1.060320000000	-3.320704000000
C	-0.078752000000	-1.108653000000	-3.219575000000
C	0.103706000000	-0.653749000000	-1.905901000000
C	0.338835000000	-1.311852000000	-0.596010000000
N	0.078419000000	0.699984000000	-2.019875000000
C	-0.146468000000	2.434467000000	-3.765019000000
N	0.000660000000	3.374959000000	-2.811162000000
C	-0.341208000000	2.763985000000	-5.113005000000
C	-0.039988000000	4.660634000000	-3.163572000000
C	-0.379379000000	4.103175000000	-5.463302000000
C	-0.226119000000	5.078478000000	-4.474700000000
H	-0.459624000000	1.972070000000	-5.844698000000
H	0.083147000000	5.378970000000	-2.356515000000
H	-0.528740000000	4.390516000000	-6.500592000000
H	-0.251069000000	6.136606000000	-4.714169000000
H	-0.589523000000	-1.698462000000	-0.157152000000
H	1.034625000000	-2.149274000000	-0.706040000000
H	0.765903000000	-0.594640000000	0.110034000000
C	-0.126756000000	-2.475893000000	-3.733975000000
C	0.026194000000	-2.726376000000	-5.120154000000
C	-0.319044000000	-3.577069000000	-2.884960000000
C	0.030598000000	-4.040903000000	-5.595330000000
C	-0.323862000000	-4.880475000000	-3.362731000000
C	-0.137904000000	-5.109704000000	-4.726874000000
H	-0.489441000000	-3.405013000000	-1.827368000000
H	0.161311000000	-4.192976000000	-6.662861000000
H	-0.478698000000	-5.709475000000	-2.678129000000
O	0.175920000000	-1.743039000000	-6.027743000000
H	0.061244000000	-0.879164000000	-5.561972000000
O	0.216478000000	1.540060000000	-0.959233000000
H	0.181323000000	2.447728000000	-1.400853000000
H	-0.136622000000	-6.124357000000	-5.116926000000

Table S10. Optimized geometry of the **S_0 state** of **$L^{OH,OH}$** (***T,N*-$L^{H,OH}$ form**, $S_0^{T,N}$) in Cartesian (XYZ) coordinates as calculated in Gaussian at the PBE0/6-31+g(d) level of theory **in the gas phase**.

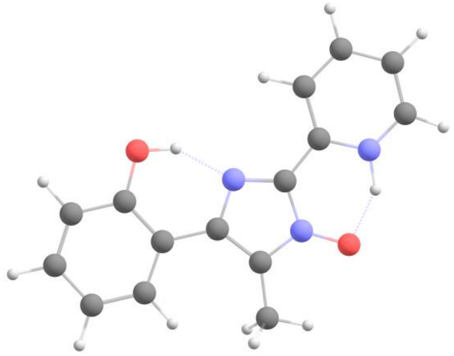

N	-0.004844000000	-0.517430000000	-0.005024000000
C	1.142560000000	0.179694000000	-0.001537000000
C	-1.006920000000	0.389319000000	-0.002891000000
C	-0.464287000000	1.703048000000	0.002722000000
C	-1.032469000000	3.072657000000	0.011401000000
N	0.877241000000	1.532694000000	0.003145000000
C	2.456834000000	-0.330783000000	-0.002765000000
N	3.475405000000	0.573466000000	0.001690000000
C	2.785487000000	-1.703919000000	-0.008239000000
C	4.771695000000	0.218993000000	0.001128000000
C	4.108621000000	-2.077697000000	-0.008797000000
C	5.133385000000	-1.105518000000	-0.004020000000
H	1.979406000000	-2.428594000000	-0.012089000000
H	5.486160000000	1.035110000000	0.004948000000
H	4.368868000000	-3.132470000000	-0.013031000000
H	6.180294000000	-1.385634000000	-0.004396000000
H	-1.637806000000	3.268192000000	-0.881477000000
H	-1.662040000000	3.245508000000	0.891932000000
H	-0.205117000000	3.787661000000	0.030831000000
C	-2.401904000000	-0.040679000000	-0.004334000000
C	-2.727753000000	-1.422333000000	0.008793000000
C	-3.462810000000	0.880690000000	-0.018307000000
C	-4.067074000000	-1.824872000000	0.010314000000
C	-4.789281000000	0.475174000000	-0.017408000000
C	-5.089589000000	-0.888502000000	-0.002407000000
H	-3.241906000000	1.941492000000	-0.031242000000
H	-4.273869000000	-2.891144000000	0.021250000000
H	-5.583130000000	1.216492000000	-0.028685000000
O	-1.796391000000	-2.391081000000	0.020294000000
H	-0.906107000000	-1.953366000000	0.013024000000
O	1.789217000000	2.475297000000	0.007725000000
H	-6.124285000000	-1.221908000000	-0.001277000000
H	3.103330000000	1.572186000000	0.005465000000

Table S11. Optimized geometry of the **S_1 state** of $L^{OH,OH}$ (***T,N***-$L^{H,OH}$ **form**, $S_1^{T,N}$) in Cartesian (XYZ) coordinates as calculated in Gaussian at the PBE0/6-31+g(d) level of theory **in the gas phase**.

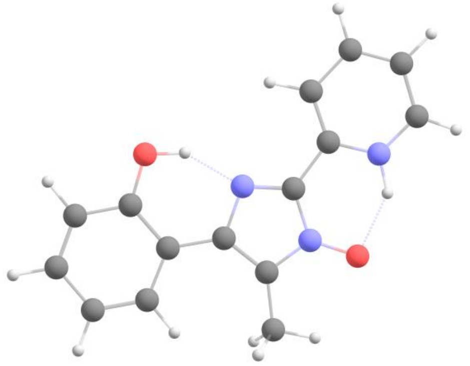

N	-0.046645000000	-0.060188000000	-4.012483000000
C	-0.040176000000	1.007716000000	-3.267741000000
C	-0.045876000000	-1.161106000000	-3.149734000000
C	-0.038404000000	-0.705035000000	-1.831728000000
N	-0.034964000000	0.662279000000	-1.923130000000
C	-0.037820000000	2.394104000000	-3.795981000000
N	-0.024984000000	3.340152000000	-2.846035000000
C	-0.046742000000	2.706979000000	-5.125019000000
C	-0.020068000000	4.685736000000	-3.138864000000
C	-0.041622000000	4.120284000000	-5.485092000000
C	-0.028550000000	5.062137000000	-4.494553000000
H	-0.056710000000	1.923922000000	-5.869648000000
H	-0.009346000000	5.368375000000	-2.302126000000
H	-0.048073000000	4.410237000000	-6.529965000000
H	-0.024454000000	6.121618000000	-4.734434000000
C	-0.034067000000	-1.349219000000	-0.495778000000
H	-0.923192000000	-1.971106000000	-0.339194000000
H	0.852952000000	-1.976103000000	-0.347365000000
H	-0.028362000000	-0.561897000000	0.263224000000
C	-0.052271000000	-2.487713000000	-3.700205000000
C	-0.059362000000	-2.699921000000	-5.120042000000
C	-0.051782000000	-3.632645000000	-2.870670000000
C	-0.065465000000	-4.005680000000	-5.633023000000
C	-0.057836000000	-4.909209000000	-3.391198000000
C	-0.064743000000	-5.095381000000	-4.785772000000
H	-0.046466000000	-3.499203000000	-1.795682000000
H	-0.070722000000	-4.121222000000	-6.712372000000
H	-0.057249000000	-5.766939000000	-2.725414000000
H	-0.069533000000	-6.099875000000	-5.199858000000
O	-0.060551000000	-1.706067000000	-6.004081000000
H	-0.055767000000	-0.842590000000	-5.504498000000
O	-0.028112000000	1.481879000000	-0.922972000000
H	-0.021119000000	2.991541000000	-1.875077000000

Table S12. Optimized geometry of the T_1 **state** of $L^{OH,OH}$ (***N,N***-$L^{H,OH}$ **form**, $T_1^{N,N}$) in Cartesian (XYZ) coordinates as calculated in Gaussian at the PBE0/6-31+g(d) level of theory **in the gas phase**.

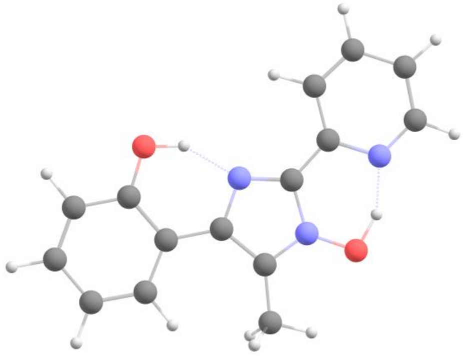

N	-0.111049000000	-0.082521000000	-4.123081000000
C	-0.058432000000	1.052595000000	-3.403157000000
C	-0.039548000000	-1.133279000000	-3.244586000000
C	0.063258000000	-0.646280000000	-1.903564000000
C	0.168558000000	-1.282055000000	-0.569002000000
N	0.049573000000	0.685759000000	-2.031918000000
C	-0.093413000000	2.384351000000	-3.804770000000
N	-0.015927000000	3.330850000000	-2.778820000000
C	-0.201142000000	2.790065000000	-5.172645000000
C	-0.044526000000	4.610576000000	-3.102262000000
C	-0.227891000000	4.124475000000	-5.470653000000
C	-0.148169000000	5.078868000000	-4.415968000000
H	-0.259280000000	2.028721000000	-5.944285000000
H	0.018359000000	5.311694000000	-2.271490000000
H	-0.309175000000	4.455747000000	-6.501985000000
H	-0.166363000000	6.144930000000	-4.613446000000
H	-0.702189000000	-1.912963000000	-0.355847000000
H	1.065775000000	-1.907245000000	-0.492582000000
H	0.225294000000	-0.504944000000	0.196840000000
C	-0.072666000000	-2.481413000000	-3.723273000000
C	-0.181911000000	-2.732236000000	-5.132247000000
C	-0.001851000000	-3.601855000000	-2.859769000000
C	-0.214873000000	-4.053233000000	-5.602999000000
C	-0.035593000000	-4.892549000000	-3.340616000000
C	-0.143125000000	-5.117160000000	-4.724578000000
H	0.081201000000	-3.442165000000	-1.791879000000
H	-0.298195000000	-4.203530000000	-6.674989000000
H	0.020713000000	-5.730685000000	-2.652533000000
O	-0.255073000000	-1.759532000000	-6.033083000000
H	-0.219331000000	-0.880163000000	-5.542463000000
O	0.125169000000	1.559717000000	-1.007802000000
H	0.082857000000	2.487379000000	-1.529198000000
H	-0.170173000000	-6.133523000000	-5.108419000000

Table S13. Optimized geometry of the T_1 **state** of $L^{OH,OH}$ (***T,N***-$L^{H,OH}$ **form**, $T_1^{T,N}$) in Cartesian (XYZ) coordinates as calculated in Gaussian at the PBE0/6-31+g(d) level of theory **in the gas phase**.

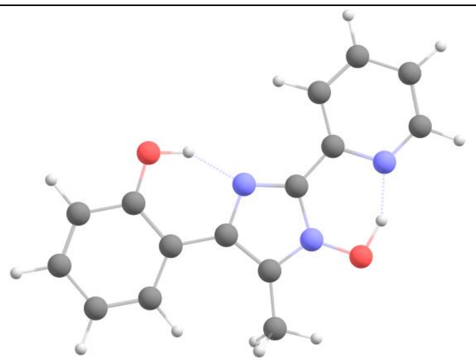

N	-0.111049000000	-0.082521000000	-4.123081000000
C	-0.058432000000	1.052595000000	-3.403157000000
C	-0.039548000000	-1.133279000000	-3.244586000000
C	0.063258000000	-0.646280000000	-1.903564000000
C	0.168558000000	-1.282055000000	-0.569002000000
N	0.049573000000	0.685759000000	-2.031918000000
C	-0.093413000000	2.384351000000	-3.804770000000
N	-0.015927000000	3.330850000000	-2.778820000000
C	-0.201142000000	2.790065000000	-5.172645000000
C	-0.044526000000	4.610576000000	-3.102262000000
C	-0.227891000000	4.124475000000	-5.470653000000
C	-0.148169000000	5.078868000000	-4.415968000000
H	-0.259280000000	2.028721000000	-5.944285000000
H	0.018359000000	5.311694000000	-2.271490000000
H	-0.309175000000	4.455747000000	-6.501985000000
H	-0.166363000000	6.144930000000	-4.613446000000
H	-0.702189000000	-1.912963000000	-0.355847000000
H	1.065775000000	-1.907245000000	-0.492582000000
H	0.225294000000	-0.504944000000	0.196840000000
C	-0.072666000000	-2.481413000000	-3.723273000000
C	-0.181911000000	-2.732236000000	-5.132247000000
C	-0.001851000000	-3.601855000000	-2.859769000000
C	-0.214873000000	-4.053233000000	-5.602999000000
C	-0.035593000000	-4.892549000000	-3.340616000000
C	-0.143125000000	-5.117160000000	-4.724578000000
H	0.081201000000	-3.442165000000	-1.791879000000
H	-0.298195000000	-4.203530000000	-6.674989000000
H	0.020713000000	-5.730685000000	-2.652533000000
O	-0.255073000000	-1.759532000000	-6.033083000000
H	-0.219331000000	-0.880163000000	-5.542463000000
O	0.125169000000	1.559717000000	-1.007802000000
H	0.082857000000	2.487379000000	-1.529198000000
H	-0.170173000000	-6.133523000000	-5.108419000000

Table S14. Optimized geometry of the T_1 state of $L^{OH,OH}$ (***N,T***-$L^{H,OH}$ **form**, $T_1^{N,T}$) in Cartesian (XYZ) coordinates as calculated in Gaussian at the PBE0/6-31+g(d) level of theory **in the gas phase**.

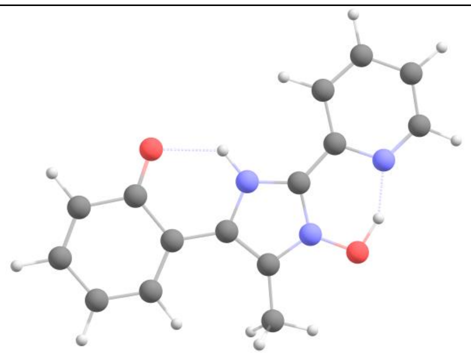

N	-0.107298000000	-0.070292000000	-4.076267000000
C	-0.056692000000	1.095212000000	-3.381974000000
C	-0.037604000000	-1.167452000000	-3.220463000000
C	0.061552000000	-0.653419000000	-1.925845000000
C	0.167501000000	-1.278235000000	-0.584862000000
N	0.047566000000	0.698731000000	-2.056656000000
C	-0.093356000000	2.426239000000	-3.804208000000
N	-0.016932000000	3.365049000000	-2.787525000000
C	-0.200607000000	2.830744000000	-5.167879000000
C	-0.045523000000	4.651629000000	-3.107769000000
C	-0.227164000000	4.169928000000	-5.460096000000
C	-0.148470000000	5.123199000000	-4.413647000000
H	-0.259857000000	2.085704000000	-5.956775000000
H	0.017563000000	5.345756000000	-2.271412000000
H	-0.308447000000	4.498349000000	-6.492802000000
H	-0.167043000000	6.188783000000	-4.613244000000
H	-0.703413000000	-1.906804000000	-0.366799000000
H	1.065614000000	-1.901123000000	-0.503895000000
H	0.223835000000	-0.495115000000	0.174324000000
C	-0.072108000000	-2.504253000000	-3.714081000000
C	-0.184502000000	-2.745221000000	-5.165793000000
C	-0.002156000000	-3.621878000000	-2.858985000000
C	-0.215471000000	-4.116203000000	-5.614750000000
C	-0.036511000000	-4.913983000000	-3.348346000000
C	-0.144152000000	-5.163748000000	-4.738790000000
H	0.080877000000	-3.474889000000	-1.788797000000
H	-0.298606000000	-4.263914000000	-6.687437000000
H	0.020037000000	-5.748180000000	-2.654539000000
O	-0.252411000000	-1.808200000000	-6.003079000000
O	0.125721000000	1.563351000000	-1.015321000000
H	0.084492000000	2.488580000000	-1.512994000000
H	-0.169407000000	-6.187975000000	-5.101415000000
H	-0.185094000000	-0.230064000000	-5.080413000000

Table S15. Optimized geometry of the T_1 **state** of $L^{OH,OH}$ (***T,T***-$\mathbf{L^{H,OH}}$ **form**, $T_1^{T,T}$) in Cartesian (XYZ) coordinates as calculated in Gaussian at the PBE0/6-31+g(d) level of theory **in the gas phase**.

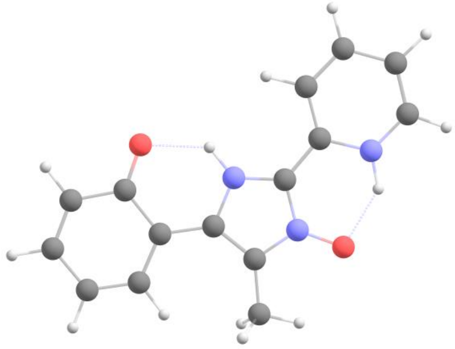

N	-0.102192000000	-0.064340000000	-4.011442000000
C	-0.049785000000	1.072301000000	-3.294203000000
C	-0.034326000000	-1.170656000000	-3.178261000000
C	0.065388000000	-0.672432000000	-1.877635000000
C	0.170904000000	-1.306098000000	-0.543548000000
N	0.054126000000	0.695287000000	-1.971688000000
C	-0.090267000000	2.404168000000	-3.765805000000
N	-0.019116000000	3.421875000000	-2.811789000000
C	-0.196418000000	2.772457000000	-5.118776000000
C	-0.050463000000	4.729866000000	-3.166598000000
C	-0.228856000000	4.098020000000	-5.487201000000
C	-0.152988000000	5.109240000000	-4.472706000000
H	-0.252872000000	1.993994000000	-5.874674000000
H	0.011100000000	5.438196000000	-2.347217000000
H	-0.310860000000	4.374683000000	-6.532783000000
H	-0.175757000000	6.162852000000	-4.726525000000
H	-0.700461000000	-1.932983000000	-0.320503000000
H	1.070160000000	-1.927404000000	-0.457879000000
H	0.226273000000	-0.508243000000	0.202218000000
C	-0.071871000000	-2.494044000000	-3.710220000000
C	-0.184786000000	-2.699946000000	-5.166439000000
C	-0.003783000000	-3.627959000000	-2.879951000000
C	-0.218212000000	-4.060085000000	-5.646539000000
C	-0.040398000000	-4.909768000000	-3.398191000000
C	-0.148567000000	-5.127080000000	-4.793749000000
H	0.079527000000	-3.499281000000	-1.806996000000
H	-0.301725000000	-4.184123000000	-6.722209000000
H	0.014717000000	-5.759716000000	-2.723689000000
O	-0.251266000000	-1.742863000000	-5.982608000000
O	0.129772000000	1.526199000000	-0.968735000000
H	-0.175672000000	-6.142724000000	-5.179727000000
H	0.057196000000	3.084708000000	-1.840701000000
H	-0.180127000000	-0.215605000000	-5.019900000000

Table S16. Optimized geometry of $\mathbf{L}^{\mathbf{OH,OH}}$ near S_0/S_1 conical intersection in Cartesian (XYZ) coordinates as calculated in Gaussian at the PBE0/6-31+g(d) level of theory **in the gas phase**.

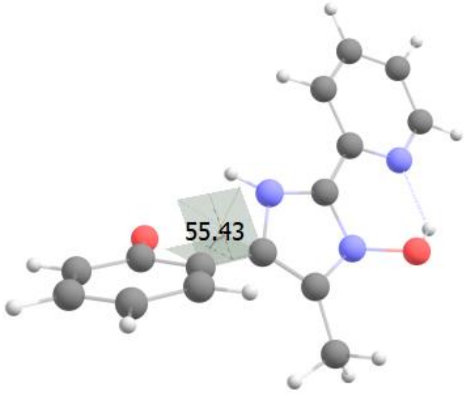

N	-0.150139000000	-0.078830000000	-4.036393000000
C	0.007242000000	1.082177000000	-3.339741000000
C	0.105821000000	-1.146964000000	-3.177735000000
C	0.400496000000	-0.667104000000	-1.955045000000
C	0.736982000000	-1.332350000000	-0.674497000000
N	0.398285000000	0.747387000000	-2.065253000000
C	-0.190429000000	2.412980000000	-3.779466000000
N	0.146362000000	3.384118000000	-2.877484000000
C	-0.683025000000	2.727860000000	-5.068301000000
C	0.025827000000	4.649088000000	-3.257088000000
C	-0.797734000000	4.054296000000	-5.427818000000
C	-0.429450000000	5.051786000000	-4.512789000000
H	-0.976821000000	1.935683000000	-5.750955000000
H	0.306580000000	5.393305000000	-2.513142000000
H	-1.175515000000	4.322274000000	-6.410711000000
H	-0.507447000000	6.105218000000	-4.758691000000
H	-0.047994000000	-1.207505000000	0.082987000000
H	0.887525000000	-2.401419000000	-0.844668000000
H	1.658463000000	-0.908240000000	-0.260830000000
C	-0.024152000000	-2.531177000000	-3.702255000000
C	0.743368000000	-2.893100000000	-4.880554000000
C	-0.912073000000	-3.419621000000	-3.152890000000
C	0.572788000000	-4.230888000000	-5.389496000000
C	-1.029350000000	-4.735337000000	-3.662551000000
C	-0.278896000000	-5.128395000000	-4.774136000000
H	-1.514865000000	-3.113516000000	-2.301092000000
H	1.165549000000	-4.508582000000	-6.256316000000
H	-1.704050000000	-5.438476000000	-3.184026000000
O	1.490984000000	-2.051432000000	-5.435343000000
H	0.160173000000	-0.165753000000	-5.007068000000
O	-0.158765226215	1.437018356216	-0.986217212986
H	0.084231226215	2.343513643784	-1.207874787014
H	-0.379106000000	-6.139621000000	-5.159629000000
